# Ectopic expression of amaranth seed storage albumin modulates photoassimilate transport and nutrient acquisition in sweetpotato

**DOI:** 10.1038/srep25384

**Published:** 2016-05-05

**Authors:** Shubhendu Shekhar, Lalit Agrawal, Divya Mishra, Alak Kumar Buragohain, Mullath Unnikrishnan, Chokkappan Mohan, Subhra Chakraborty, Niranjan Chakraborty

**Affiliations:** 1 National Institute of Plant Genome Research, Jawaharlal Nehru University Campus, Aruna Asaf Ali Marg, New Delhi-110067, India; 2 Department of Molecular Biology and Biotechnology, Tezpur University, Assam, India; 3 Central Tuber Crops Research Institute, Thiruvananthapuram, Kerala, India

## Abstract

Storage proteins in plants, because of high nutrient value, have been a subject of intensive investigation. These proteins are synthesized *de novo* in the cytoplasm and transported to the storage organelles where they serve as reservoir of energy and supplement of nitrogen during rapid growth and development. Sweetpotato is the seventh most important food crop worldwide, and has a significant contribution to the source of nutrition, albeit with low protein content. To determine the behaviour of seed storage proteins in non-native system, a seed albumin, AmA1, was overexpressed in sweetpotato with an additional aim of improving nutritional quality of tuber proteins. Introduction of AmA1 imparted an increase in protein and amino acid contents as well as the phytophenols. The proteometabolomics analysis revealed a rebalancing of the proteome, with no significant effects on the global metabolome profile of the transgenic tubers. Additionally, the slower degradation of starch and cellulose in transgenic tubers, led to increased post-harvest durability. Present study provides a new insight into the role of a seed storage protein in the modulation of photoassimilate movement and nutrient acquisition.

Storage organs in plants display diverse nutritional excellence and complex multistep development, and serve as sinks for nutrients. Storage proteins have tissue-specific expression and are the most abundant proteins in storage tissues. They serve as a nutritional reservoir by providing an indispensable source of carbon, nitrogen and amino acids during growth and development, particularly during seed germination until the establishment of photosynthetic capability in the developing seedlings. The composition of nutrients in storage organs, particularly carbon and nitrogen, greatly influence the development and nutritional quality of the organ^1^,^2^. Seed storage proteins contain nutritionally significant amounts of all essential amino acids. Furthermore, they play a crucial role in productivity, and are duly acknowledged for their dual role in plant growth and as an essential component of human diet^3^,^4^,^5^. Comprehensive investigation pertaining to the isolation and characterization of seed storage proteins not only has improved our understanding of their biogenesis and genetic control, but also paved the way for targeted manipulation of crops for nutritional improvements. Seed storage proteins are regulated via an integrated genetic and physiological network. The crosstalk between the underlying developmental program and their genetic and physiological regulation, to a greater extent, is permutated by extrinsic conditions. Besides these conditions, the nutrient source flux and the nutrient sink development eventually dictate the ultimate content of mature seeds. Moreover, outcome of the seed genetic program in developing the sink is greatly modulated by the nutrient status of the maternal plant, which provides additional regulatory control. During this genetic program, nutrient source is directly coupled with the nutrient sink^6^,^7^,^8^. Despite decades of research, particularly on genetic control of spatiotemporal regulation of seed storage proteins, our understanding of their precise function and physiological significance remains fragmentary. In recent years, much attention has been given to investigate the transgenic crops overexpressing storage protein; however, their operational framework, and molecular and physiological consequences for perceiving a storage protein have not been elucidated^5^,^8^,^9^.

Tuber crops have an enormous agronomical importance for food security and source of income for 2.2 billion people in developing countries^10^. In addition to the nutritional benefits, the adaptability of sweetpotato to a broad range of agro-ecological conditions with minimal growth requirements, makes it a favorable crop of high commercial significance^11^,^12^. All genotypes of sweetpotato, to a varying degree, are an exceptionally good source of vitamins, dietary fiber and several micronutrients, in addition to very low content of fat and cholesterol. However, the protein content of most genotypes is reported to be low^13^,^14^. During past decades, several efforts have been made to improve the protein quality and quantity in sweetpotato but with very limited success^15^,^16^.

In recent past, a number of potential candidate genes associated with nutrient acquisition have been targeted to improve the nutritional quality of crops, particularly the protein content. However, the overexpression of such genes in target crops frequently resulted in the augmentation of particular amino acids at the expense of others, causing a disparity in the amino acid profile of the transgenics^17^. Notably, the amaranth seed albumin AmA1 has huge agricultural significance with a well balanced composition of amino acids that is superior even to that recommended by the WHO for a nutritionally rich protein^18^. While sweetpotato proteins are deficient in tryptophan and sulphur amino acids^19^, the AmA1 is rich in all essential amino acids^17^. To determine the expression of AmA1 in an alien environment, particularly in a tuberous root, and to examine its functional diversity, the AmA1 was introduced into sweetpotato through genetic engineering. This strategy to improve sweetpotato may prove to be more acceptable to the community compared with those presently used in transgenic crops because the *AmA1* gene is an edible crop derived sequence.

## Results

### Stable integration and expression of seed storage protein

**Figure 1:**
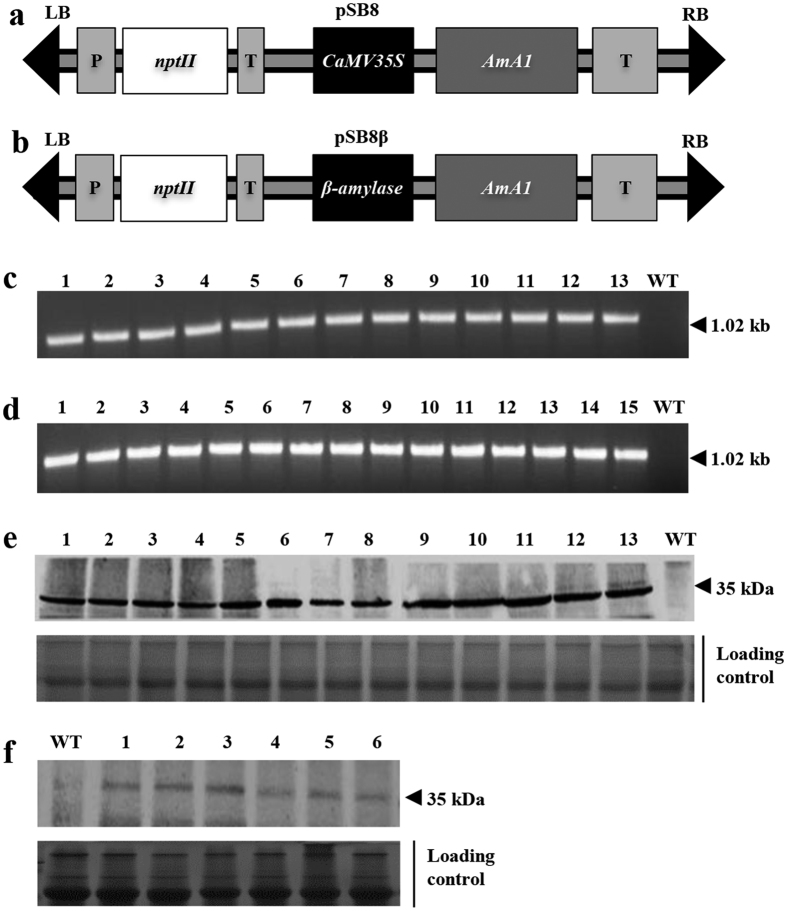
Integration and expression of *AmA1*. Schematic representation of expression vectors pSB8 (**a**) and pSB8β (**b**) harbouring *AmA1* cDNA under the control of CaMV *35S* and β-amylase promoters, respectively. LB, T‐DNA left border; P, *NOS* promoter; T, *NOS* terminator; RB, T‐DNA right border; *nptII*, neomycin phosphotransferase II. Integration of *AmA1* gene was confirmed by PCR analysis in COE (**c**) and TOE (**d**) lines. An amplicon of 1.02 kb confirmed transgene integration in putative transformants. Lane numbers are representative of the individual transgenic lines, while WT corresponds to an untransformed plant. Immunodetection of AmA1 in COE (**e**) and TOE (**f**) lines. CBB-stained gel as loading control is shown in the lower panel of each blot.

Genetic transformation of sweetpotato was carried out using two different *AmA1* constructs (Fig. 1a,b) by Agrobacterium-mediated transformation as described previously^20^. The integration of *AmA1* was examined by PCR at the genome level in all kanamycin-selected putative transformants. Of the 18 kanamycin-resistant plants, constitutively overexpressed (COE) *AmA1*, 13 were true transformants, as revealed by 1.02 kb amplicon, which was otherwise absent in the wild-type (WT). Furthermore, all of the 15 kanamycin-resistant plants, tuber-specifically overexpressed (TOE) *AmA1*, were positive (Fig. 1c,d). Additionally, qRT-PCR analysis revealed a low copy number of the introduced transgene and presence of a single copy of the *AmA1* in most transgenic lines.

### Immunoblot analysis

A 35 kDa band was identified by immunodetection of AmA1 in tubers of both COE and TOE transgenic lines, but at varying levels, suggesting that AmA1 was a functional protein in an alien system (Fig. 1e,f). The band intensity corresponding to AmA1 was quantified, and the transgenic lines were categorized into three groups, *i.e*., low, moderate and high expression. One each from the low and moderate expression group, and 4 from the high expression group of both COE and TOE lines were selected for further analysis.

### Transcript abundance and spatiotemporal expression of transgene

**Figure 2:**
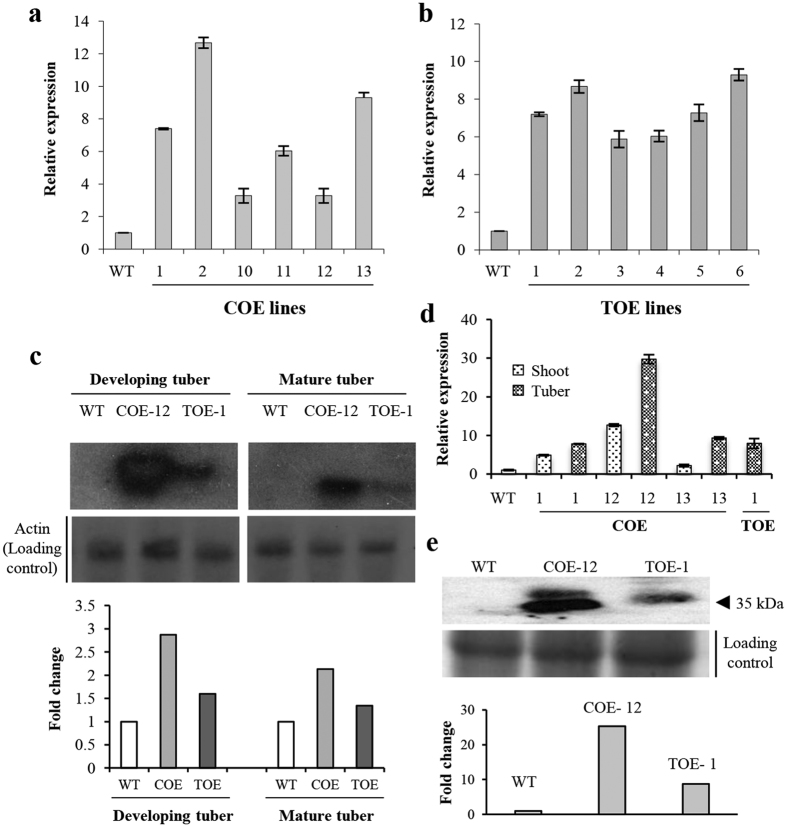
*AmA1* transcript abundance. The *AmA1* transcript abundance was analyzed in COE (**a**) and TOE (**b**) lines by qRT-PCR using *actin* as endogenous reference gene. Numerals on X-axis represent individual lines. Transcript abundance was also evaluated by northern blot in developing and mature tubers of COE-12 and TOE-1, and compared with that of WT (**c**). β-actin served as loading control. The autoradiogram was quantified by Quantity One software. Further, *AmA1* transcript abundance in the shoots and tubers of COE lines was analyzed by qRT-PCR and compared with that of TOE line (**d**). Numerals on X-axis represent individual lines. Immunodetection of AmA1 in COE-12 and TOE-1 lines (**e**). The histograms represent fold changes in expression values. CBB-stained gel served as loading control.

The transcript abundance of *AmA1* was analyzed in the selected COE and TOE lines, using *actin* as a reference gene. A high abundance of transcripts was detected in most transgenic lines compared with that in the WT. The expression analysis revealed that transcript abundance was >3- to 12-fold higher in different COE lines than in the WT, whereas the TOE lines had 5- to 9-fold higher expression (Fig. 2a,b).

Because the expression of most seed proteins is temporally and spatially regulated, we examined the spatiotemporal expression of *AmA1*. The most promising transgenic lines from both categories (COE-12 and TOE-1) were selected for this analysis. The northern blot revealed steady expression of *AmA1* until tuber maturity, with a slight decrease in mature tubers. This might be due to the intrinsic characteristics of the AmA1, because seed storage proteins act as a sink of biological reserve. Notably, the expression of *AmA1* in the TOE line was relatively lower than COE lines, particularly during the developmental stages (Fig. 2c). To validate the expression profile, *AmA1* expression in the shoots and tubers of the COE lines was compared with that in the TOE lines by qRT-PCR. The results revealed an overall induced *AmA1* expression in tubers compared with shoots of the COE lines. The TOE lines had lower expression in the tubers than that in the COE lines (Fig. 2d), which was in concordance with the northern blot analysis. Furthermore, immunoblot analysis was performed to test AmA1 expression with the same lines that were used for the northern blot and qRT-PCR analyses. The results revealed a 2.5-fold higher expression of AmA1 in the COE-12 line than TOE-1 line (Fig. 2e).

### Comparative proteomics

**Figure 3:**
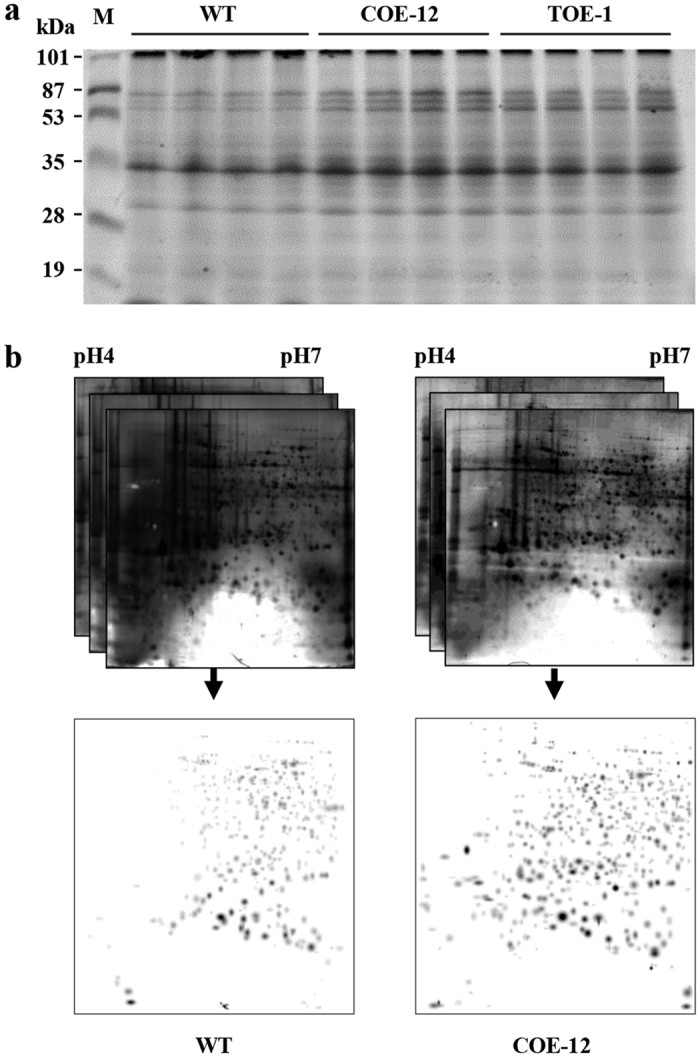
Comparative proteome profile. Proteomics analysis was performed in equal mass (2 g) of mature tuber, and aliquots of 100 and 250 μl proteins were separated by 1-DE (**a**) and 2-DE (**b**), respectively. Three replicate 2-DE gels from WT and COE-12 were independently combined using PDQuest software (version 7.2.0) to generate “standard gels” as described in ‘Materials and methods’.

To evaluate the possible effects of AmA1 expression in the transgenics, a comparative proteomics analysis was performed using the proteins extracted from equal masses of tubers. An equal volume of 100 and 250 μl protein extracts was separated by 1-DE (COE-12 and TOE-1) and 2-DE (COE-12), respectively. The 1-DE analysis showed no change in the proteome profile, whereas a change in the total protein content was evident compared with that in the WT (Fig. 3a). Screening of 2-D gels revealed an increase in the number of protein spots for the transgenics (491) compared with that in the WT (467) (Fig. 3b).

**Figure 4:**
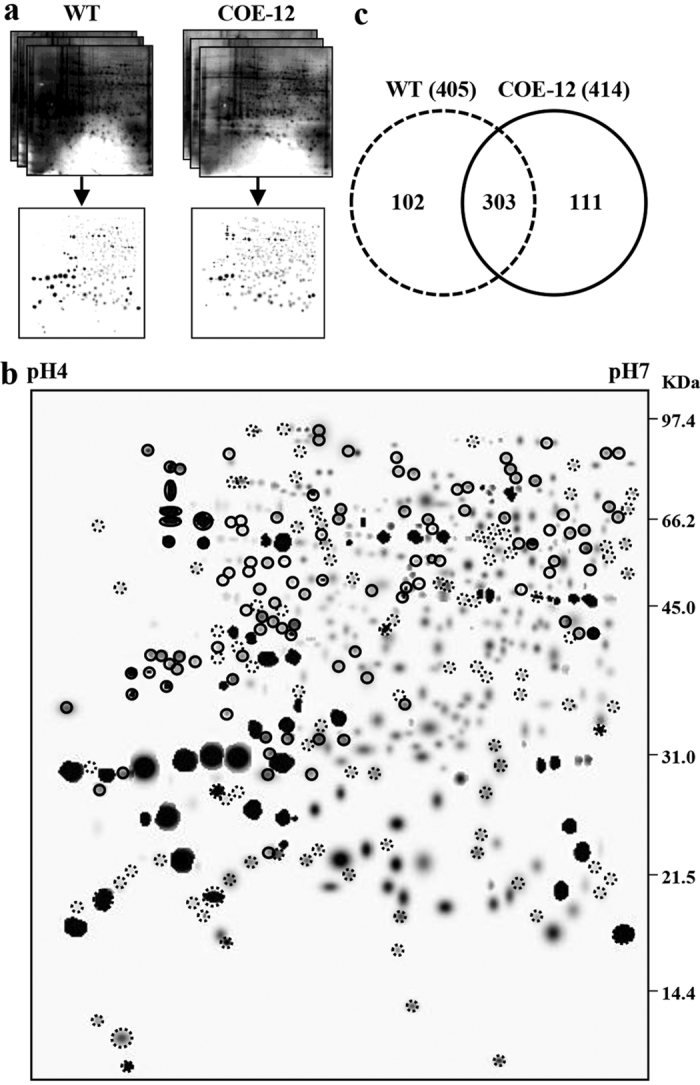
Comparative proteomics of transgene mediated change(s). The proteomes displaying three replicate gels from WT and COE-12 were computationally integrated into the “standard gel” as described in ‘Materials and methods’ (**a**). The differential proteome was developed from the “standard gels”. The exclusive protein spots are shown with dotted and solid circles for WT and COE-12 tubers, respectively (**b**). Venn diagram shows the common and exclusive proteins in AmA1 modulated alteration in proteome profiles. The areas in the diagram are not proportional to the number of proteins in each group (**c**).

To further investigate transgene-mediated change(s) in the tuber proteome, the WT and COE-12 tubers were subjected to comparative proteomics using 2-DE. The first-level match set generated from the replicates had a correlation co-efficient above 0.8, and the gels showed >90% high quality protein spots, indicating great reproducibility (Supplementary Fig. S1 and Supplementary Table S1). A second-level match set was then developed for comparison of the standard gels from both WT and COE line. A second normalization was performed, and a set of three unaltered spots was identified across the gels. Of the 405 and 414 high quality spots in WT and COE lines, respectively 303 protein spots were common to both; however, 102 spots were exclusive to the WT and 111 to the COE lines (Fig. 4). The analysis revealed several contrasting trends and commonalities as well in the proteomes.

### Determination of increased protein content in AmA1-overexpressed transgenics

**Figure 5:**
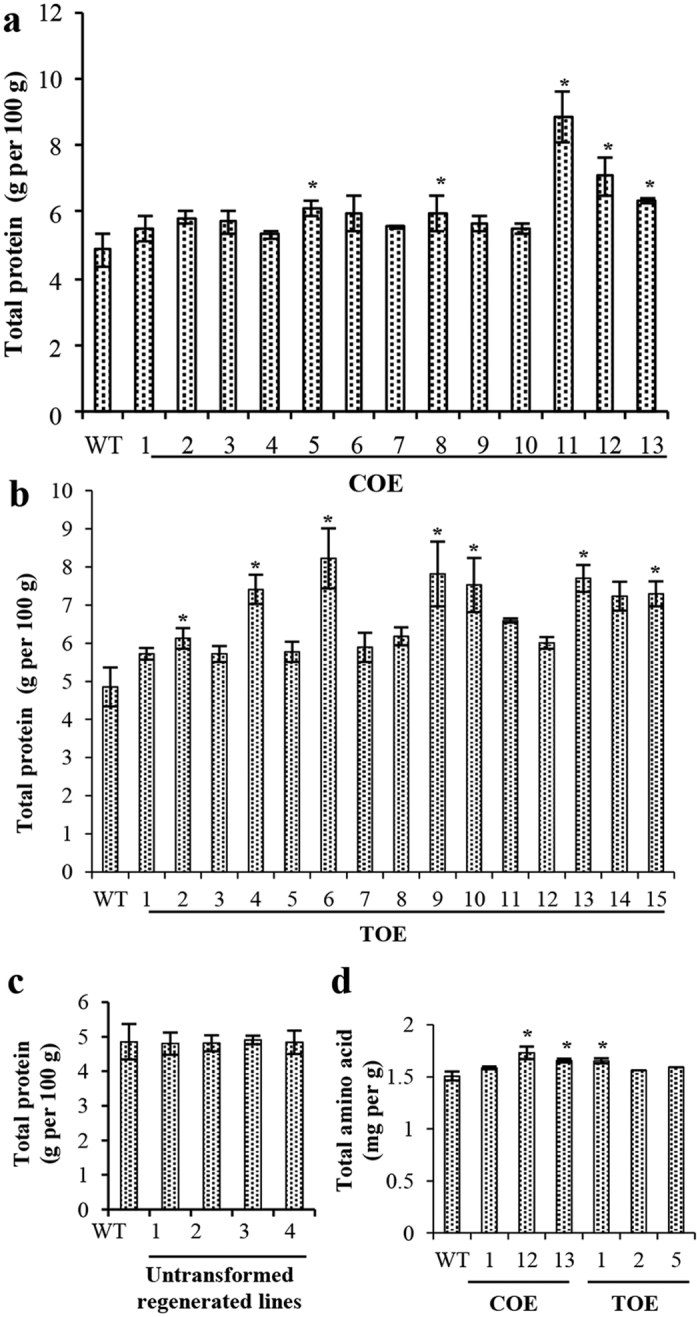
Quantitative determination of protein and amino acid contents. Protein content of WT was compared with that of COE (**a**), TOE (**b**) and untransformed plants regenerated from callus (**c**). The quantification was performed with five independent harvests. Total content of amino acids was evaluated in WT and transgenic lines by ninhydrin based calorimetric assay as detailed in ‘Materials and methods’ (**d**). Numbers on X-axis represent individual lines. Data represent mean ± s.e. of five measurements and asterisk (*) indicate the level of statistical significance at *p* < *0.05*.

The total protein content was quantified in tubers of both transgenic events and WT by micro-Kjeldahl analysis. The results revealed an increase of approximately 10–83% in the protein content of the COE lines when compared with the WT (Fig. 5a). Notably, the increase in protein content of the TOE lines was in the range of 18–70%, which corroborated higher transgene expression in the COE than in the TOE lines (Fig. 5b). To test whether there was an effect of micropropagation on the increase in protein content, tubers of untransformed plants regenerated from callus were examined, and no significant differences were found when compared with the WT (Fig. 5c).

**Figure 6:**
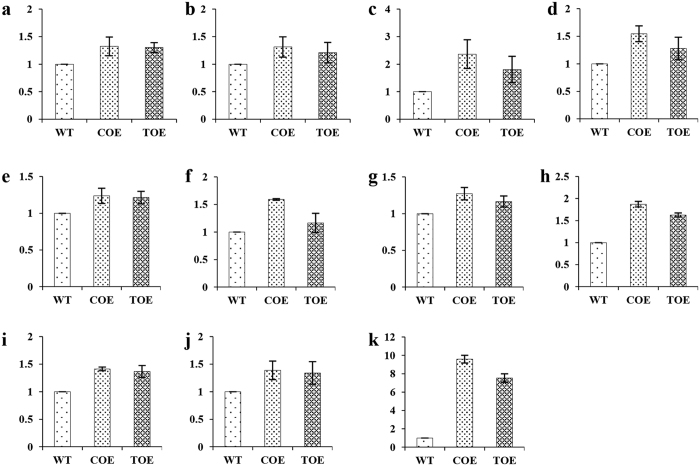
Comparative analysis of amino acids by GC-MS. Amino acids of WT were compared with that of transgenic tubers. Amino acids L-leucine (**a**), serine (**b**), valyl-valine (**c**), L-aspartic acid (**d**), phenylalanine (**e**), L-asparagine (**f**), alanine (**g**), glutamine (**h**), L-lysine (**i**), L-valine (**j**) and L-methionine (**k**) are shown. Data were normalized to the mean response calculated for WT levels of each replicate that allowed comparison among the replicates. Ribitol served as internal standard. Y-axis represents relative response ratio across WT and transgenic lines (COE-12 and TOE-1). Data represent the mean ± s.e.

The nitrogen content in dietary protein is approximately 16% by weight, and nitrogen metabolism is often considered to be tantamount to protein metabolism. The increase in protein content of the transgenic lines directed to study the transgene effect at amino acid level. The amino acids were analyzed with a ninhydrin-based calorimetric method, which revealed an increase in the free amino acid pool size in the transgenics (Fig. 5d). The absolute quantification of the metabolites was accomplished by GC-MS analysis. The only amino acids that were considered were those that co-eluted at or approximately the same retention time. The analysis revealed a significant increase in the content of several amino acids, notably, lysine, leucine, methionine, phenylalanine and valine, which are otherwise limited in sweetpotato. An increase in the levels of non-essential amino acids, such as alanine, aspartic acid, glutamine, asparagine and serine further indicated that the augmentation of essential amino acids in the transgenics was not at the expense of other amino acids (Fig. 6).

### Assessment of the agrophysiological traits

**Figure 7:**
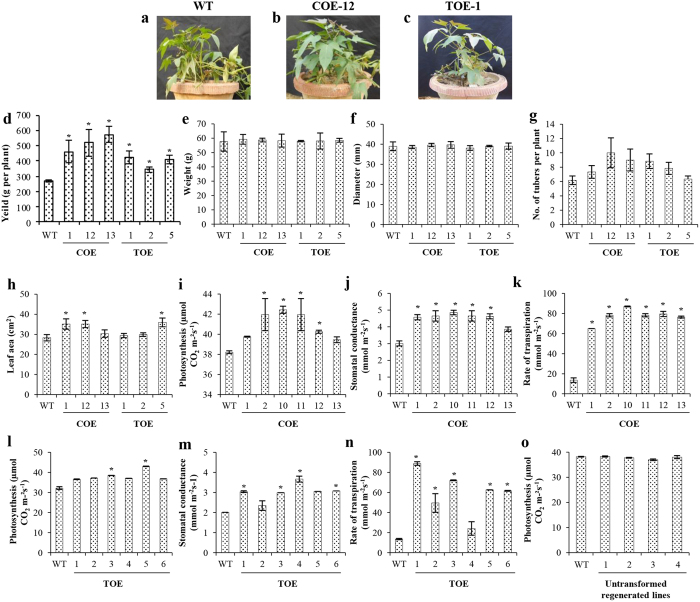
Comparative analysis of agrophysiological traits. Representative photographs displaying the morphology of WT **(a)**, COE (**b**) and TOE (**c**) transgenic lines. Differences in yield (**d**), weight (**e**), diameter (**f**) and number of tubers per plant (**g**) were analyzed across the WT and transgenic lines. Comparative analyses of leaf area (**h**), photosynthetic rates (**i,l**), stomatal conductance (**j,m**) and rate of transpiration (**k,n**) were carried out for WT and transgenic lines (COE and TOE). Net photosynthetic activities in untransformed plants were also measured (**o**). Each bar indicates the mean values ± s.e. (n = 5) and asterisk (*) indicates the level of statistical significance at *p* < *0.05*.

The transgenic plants were grown in parallel with the WT in identical experimental conditions, and the plants showed no morphological differences or constraints to growth. The average yield was significantly higher for most transgenic events, although the average tuber weight and diameter were not affected. By contrast, the average number of tubers per plant increased considerably in transgenic plants, which might have contributed to the higher harvest index (Fig. 7a–g).

Because leaf area is a primary determinant of photosynthetic efficiency and productivity, we determined the leaf area profiles and photosynthetic CO_2_ fixation. A positive correlation between the increased leaf area and photosynthetic efficiency was observed in both the COE and TOE lines (Fig. 7h,i,l). The photosynthetic CO_2_ fixation, stomatal conductance and rate of transpiration, which are interlinked, were also higher in both the COE (Fig. 7j,k) and TOE (Fig. 7m,n) lines. Nevertheless, the photosynthetic efficiency of regenerated untransformed plants was comparable with that in the WT (Fig. 7o).

### Biochemical and proximate composition

To test the AmA1-mediated changes in the fundamental metabolism and nutrient availability, extensive biochemical analyses were conducted. The proximate analysis revealed no significant differences in moisture, ash and fiber contents between the WT and transgenic lines (Supplementary Table S2). A comparable amount of carbohydrates but a higher reducing sugar content was observed in the WT tubers than in the transgenic lines. Notably, the reducing sugar content was reduced by up to 50% in the transgenic lines. However, the starch content increased in both COE and TOE lines (Supplementary Fig. S2a–c).

### Quantitative determination of water holding capacity (WHC)

The WHC is the quantity of water retained by a known amount of sample, and is reasonably correlated with protein and carbohydrate, particularly the starch content^14^. A direct correlation was observed between the starch and protein content as the WHC was higher in the AmA1-tubers than the WT (Supplementary Table S2).

### Storage stress response in tubers

To verify the disparity in carbohydrate content, the degradation patterns of starch and cellulose were assessed in the WT and transgenic lines during post-harvest storage. The starch content progressively declined until 105-d, and an approximately 29% loss was observed in the WT. The rate of starch degradation was comparatively slower in the transgenic lines. The decline was 23% in the COE-12, whereas 26% in the TOE-1 line (Supplementary Fig. S2d). The alteration in cellulose content showed a similar trend, whereas the cellulose content in the WT decreased progressively, and the degradation in the transgenic lines was less prominent. The degradation of cellulose was 35% in the WT and 31% and 32%, respectively, in the COE-12 and TOE-1 (Supplementary Fig. S2e).

### Structural and chemical characterization

To investigate whether expression of AmA1 caused any structural modification of starch, SEM analysis was conducted on composite samples of the transgenics and WT tubers. The crystal structure of starch did not show any detectable modification in transgenic plants. These findings were consistent with the FT-IR spectra, which accurately identify the presence of functional groups in the chemical structure of the composite samples. A few characteristic absorption bands were observed (Supplementary Fig. S3), notably, the C–O, C–H and hydroxyl groups-stretching vibrations. Compared with the WT samples, the AmA1-tubers showed no additional spectra, which negate any structural modification of the starch.

### Colour difference in tubers, and determination of phytophenols and carotenoids

The influence of AmA1 expression on tuber flesh colour was evaluated by comparative colour difference. The Hunter ‘L’, ‘a’, and ‘b’ colour measurements were consistent with the visual appearance of tuber flesh (Supplementary Fig. S4a–c). The colour space values indicated that the colour were distinct in the WT and AmA1-tubers. No significant change in the ‘L’ (white) value was observed in the COE and TOE lines. A marked increase in the ‘a’ (red) values was observed in most transgenic events; the ‘a’ value increased up to 4.2- and 1.6-fold in the COE-12 and TOE-1 lines, respectively. However, the difference in the ‘b’ (yellow) values was not significant in the transgenics compared with the WT (Supplementary Fig. S4d–f).

Sweetpotatoes are a rich source of anti-oxidants, particularly phytophenolics and carotenoids, which contribute to the distinct flesh colour^10^,^14^. Because differences were observed in the tuber flesh colour, detailed biochemical analyses were conducted which revealed contrasting levels of phytophenols and carotenoids. The increase in the total phenolic content (TPC) and total flavonoid content (TFC) was clearly evident in the transgenic lines. Because anthocyanin is among the essential groups of phenolic compounds, the total anthocyanin content (TAC) of both the WT and transgenic lines was expected to follow a similar trend to that of the TFC. The TAC of the transgenic lines was significantly higher than that in the WT. However, the total carotenoid content (TCC) in most transgenic lines was not significantly different from that in the WT, except in the high AmA1-expressing lines (Supplementary Fig. S5).

### Differential expression of flavonoid pathway genes

Because AmA1-tubers displayed an increase in the level of phytophenols, the relative transcript accumulation of key flavonoid pathway genes viz., chalcone synthase (*CHS*), chalcone flavanone isomerase (*CHI*), flavanone 3-hydroxylase (*F3H*), dihydroflavonol 4-reductase (*DFR*), anthocyanidin synthase (*ANS*) and UDP-glucose flavonoid 3-O-glucosyl transferase (*UFGT*), was examined. The transcript analysis revealed a high abundance of early pathway genes *CHS* and *CHI* (Supplementary Fig. S6a,b) in addition to the late pathway genes *DFR, ANS* and *UFGT*, in AmA1-tubers (Supplementary Fig. S6d–f).

### Comparative metabolome profiling and allocation of metabolites

The metabolome profiling of tubers from the WT and two independent transgenic lines, one each from the COE-12 and TOE-1 lines, was performed by GC-MS. The metabolome profiling led to the identification of 153 metabolites in the WT out of which 123 were nonredundant. However, 122 and 121 metabolites were nonredundant out of 150 and 135 in the COE and TOE lines, respectively (Supplementary Table S3). In a comparison of nonredundant metabolites, 101 were common across the WT and transgenic lines, whereas 9 were specific to the transgenic lines. Compared with the metabolites of the WT, 8 and 1 metabolite was common in the COE and TOE lines, respectively (Supplementary Fig. S7). Nevertheless, 13 metabolites were unique to the WT, 4 to the COE and 10 to the TOE lines. Notably, the identified metabolites had no reports that associated them with toxicity or allergenic properties, and were the derivatives of natural compounds.

## Discussion

To understand the source and/or sink regulation of seed storage protein in an alien system, particularly in root tubers, AmA1 was overexpressed in sweetpotato. We combined proteogenomics and proteometabolomics approaches to explore the genetic switch that dictated the overall tuber development, expression of developmental gene(s), and accumulation of reserve substances. The objective was also to improve the nutritional quality of sweetpotatoes, particularly the protein content. To ensure the constitutive and tuber-specific expression, the *AmA1* was placed under *CaMV35S* and *β-amylase* promoters, respectively. The transcript analysis revealed a range of 3- to 12-fold higher expression of *AmA1* in different COE and TOE lines when compared with WT (Fig. 2a,b). To examine the developmentally regulated expression and stability of *AmAl*, a comparative analysis of the transgenic lines was conducted. The northern blot analysis revealed higher transcript abundance in the COE than the TOE lines. The transcript abundance was reexamined with qRT-PCR, which revealed tuber-specific expression of AmA1 in the TOE lines with no leaky expression (Supplementary Fig. S8); leaky expression in organs or tissues often results in unexpected phenotypes^21^,^22^.

Next we determined whether there was any metabolic shift caused by *AmA1* integration using a multivariate analysis. The transgenics had higher yields than the WT plants (Fig. 7d). The increased tuber yield was closely related with the photosynthetic carbon metabolism, a crucial factor for plant growth and productivity^23^. Therefore, we investigated the photosynthetic efficiency of the transgenics *vis-a-vis* the WT plants, which revealed superior efficiency in the transgenic lines. A strong correlation was observed between the increased leaf areas and photosynthetic rate (Fig. 7), along with increased protein content (Figs 3 and 5). The increase in protein biosynthesis might occur because of an increased rate of photosynthesis, which is eventually the crucial factor for higher yield^5^,^17^. The source-sink relationship in plants, particularly the capacity of tuberous root to accommodate assimilates, is the deciding factor in determination of total dry matter^24^. The storage organs such as tubers are sinks that compete for the available photoassimilates, and increased harvest index in tuber signifies an efficient diversion of photoassimilates towards the sink^25^,^26^. Transgenic crops that have changes in the source and/or sink capacities are often associated with impaired^27^ or superior yields^28^, which indicate the effect of such approaches on increase in yield. In this study, the expression of AmA1 in the COE line was high, as indicated by immunoblot analysis (Fig. 2e). Because all photoassimilates are directed towards the tuber during tuberization, the COE lines have an additional advantage of AmA1 expression than the TOE lines, even in the source tissues. Therefore, cumulative expression of AmA1 in the source and sink tissues might explain the high expression of the transgene in the COE lines. Additionally, because seed proteins have a crucial role in storage tissues, the source-directed expression of AmA1 in the tubers is natural. Unlike most seed storage proteins, which typically localize to protein bodies, AmA1 localizes in the cytoplasm, which might explain its delayed breakdown because it is apparent that proteolytic enzymes are not released into cytoplasm until the final stages of cellular disorganization^29^.

The overexpression of AmA1 in sweetpotato caused an increase in total proteins. The higher content of amino acids in the transgenics further corroborated the increase in the total proteins (Figs 5 and 6). The fact that the increase in amino acids was not restricted to a particular group contradicts several previous reports depicting the transgene induced increase in one amino acid at the expense of the others^30^,^31^,^32^. Nevertheless, the expression level of AmA1 in transgenic tubers was not sufficiently high to be directly correlated with the protein increase *per se*, although comparative proteome profiling showed a positive correlation with the findings of biochemical analyses (Fig. 3). The *de novo* synthesis and accumulation of unique proteins, quantitative changes in the expression level and rebalancing of proteins, possibly mediated by AmA1 expression could explain the increase in protein content. The synthesis of storage proteins depletes the pool of free amino acids in storage organ, which typically leads to an increase in the rate of photosynthesis^33^. It is likely that AmA1, when introduced into sweetpotato, might lead to a depletion of the endogenous free amino acids for the synthesis and subsequent accumulation of protein. Thus, the reduction of free amino acids in AmA1-tubers seems to have perceived by the photosynthetic machinery, which might lead to an elevated rate of photosynthesis^5^,^17^.

While elevated starch content was found in the transgenic tubers, no significant change was observed in total carbohydrates. Notably, the reducing sugar content was higher in the WT than in the AmA1-tubers. The disparity between starch and reducing sugar contents indicated the possible degradation of starch and cellulose with their hydrolysis into reducing sugars during storage^14^. These result prompted us to examine the degradation pattern of starch in WT and AmA1-tubers. The transgenic lines maintained constant high starch and cellulose contents during storage, and even the extent of degradation was lower in the transgenic tubers. The expression of AmA1 might have induced a more sustainable basal metabolism such that the demand was less for the degradation of stored starch and/or cellulose. Furthermore, the increase in nitrogen storage in the sink tissues was likely to have altered the total biomass production. A plausible explanation might be that the carbohydrate allocation in the AmA1-tubers was influenced by the increase in nitrogen supply, which strongly controlled the progression of carbon metabolism and caused greater efficiency in postharvest performance^34^.

Proteomic analyses of plant organs and tissues are widely used to monitor transgene-mediated changes in various crops^5^,^35^,^36^,^37^. To assess the AmA1-mediated changes in the tuber proteome, a comparative proteomics was performed on the WT and COE-12 line. The comparison revealed several proteins that were conserved and uniquely expressed, which indicated that subtle changes in the genome might lead to distinct proteome (Fig. 4). The relative distribution of seed proteins is principally genetically determined in addition to the variability caused by nutrient modulation. The storage tissues have intrinsic compositional plasticity concomitantly from the alteration of the source-sink relationship; this relationship may be disturbed by the accumulation of alien proteins as an alternative sink protein^8^,^38^. Additionally, overexpression of an extrinsic or underexpression of any intrinsic protein directs the cellular system to rebalance overall proteome. This situation is often compensated by the expression of some other proteins that lead to rebalancing of the nitrogen sink to maintain the metabolic cues at a more or less constant level. Seed storage proteins serve as the sink to regulate the movement of photosynthates into the developing organs^33^. Thus it was expected that AmA1, as a storage protein, might act as a sink protein in the transgenic plants, thereby regulating the allocation of metabolites, including amino acids. The overexpression of AmA1 in storage organs such as tubers and seeds was previously shown to regulate nutrient acquisition, which facilitated an increase in protein and amino acid content^17^,^18^,^39^. It was further demonstrated that AmA1 might play a crucial role during organogenesis and was essential for homoblastic growth and synchronization of different metabolic pathways directed towards tuber development. The AmA1-regulated protein network and its combinatorial effects may increase the protein content and determine the tuber development^5^ in addition to playing a key role during seed germination and seedling growth in the native and alien system^17^.

Metabolomics are used as the genetic footprint of the effect(s) of genes or gene-products and their interactions with the environment. Connecting the metabolomic information with the mRNA and protein expression data helps to envisage the functional genomics repertoire of an organism. Metabolomics are used to determine whether the level of substantial equivalence of transformants with that of their WT^40^,^41^. Therefore, we examined the *AmA1*-mediated changes in the metabolome of the transgenic tubers, which revealed contrasting trends in phytophenols and carotenoids. The TPC, TFC and TAC content was significantly higher in the transgenic tubers than the WT and in part, followed the trend of *AmA1* expression (Supplementary Figs S5 and S6). Polyphenols are synthesized from phenylalanine produced by the shikimate pathway, and flavonoids are produced through a bifurcation of this pathway. Therefore, an increase in the overall amino acid contents, phenylalanine in particular (Fig. 6), might be expected to increase the flux of TPC and TFC. These results were consistent with high expression of key flavonoid pathway genes in the transgenics (Supplementary Fig. S6). A comprehensive analysis of the metabolites was performed with the tubers of WT and two independent transgenic lines, which showed no major changes (Supplementary Table S3). Several global metabolome analyses previously suggested that no substantial differences occurred between transgenic and wild-types, except for the metabolites directly associated with the introduced pathway^42^.

**Figure 8:**
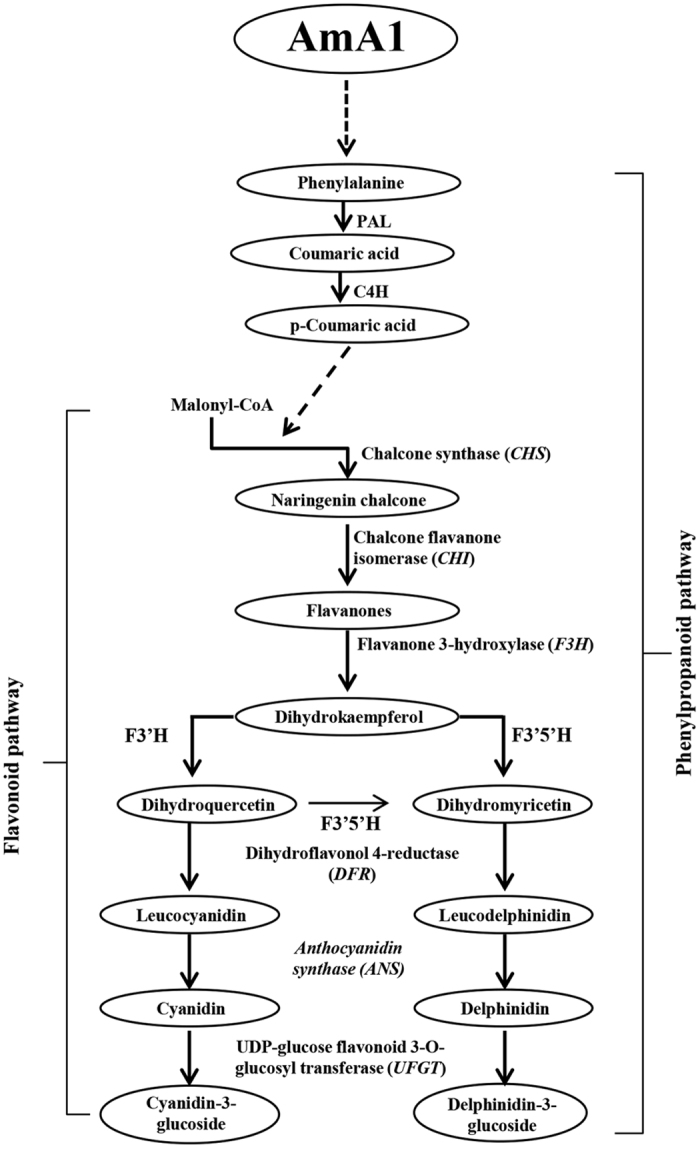
A proposed hypothetical model depicting the role of AmA1 in transgenics. Modulation of shikimate pathway via increased level of several amino acids, particularly phenylalanine, might increase the flux of TPC and TFC in the transgenics. The alteration in shikimate pathway positively correlated with increased expression of key flavonoid pathway genes. The proposed model suggests that seed albumin AmA1 might contribute to higher accumulation of phytophenols in the transgenic tubers.

Increasing evidence indicates that an increase in amino acid concentration might lead to an increase in phytophenols with the formation of three aromatic amino acids-tryptophan, tyrosine and phenylalanine, via the shikimate pathway^43^,^44^,^45^. Furthermore, these are the primary metabolites that provide the precursors for several natural secondary products, i.e., flavonoids, phenolic acids, alkaloids, glucosinolates and cyanogenic glycosides^46^. Additionally, the majority of secondary metabolites with antioxidant properties including various classes of phenolic compounds are synthesized via this pathway. Among the phytochemicals that possess antioxidant capacity, phytophenols are one of the most significant groups^47^. A high concentration of polyphenolic compounds, such as phenolic acids and flavonoids, were also reported in amaranth grains^48^, the source of *AmA1*. Because the AmA1 expression is higher in the transgenic tubers, consequently increasing the pool size of proteins and amino acids it is likely directing the sink organs for an increased synthesis of phytophenolics. Therefore, a hypothetical model is proposed (Fig. 8) for the role of AmA1 to increase the content of phenylalanine, which is consequently directed to shikimate pathway and leads to higher concentrations of phenolic acids and flavonoids.

In summary, the seed storage protein, AmA1, seems to have profound effect(s) on nutrient acquisition in sweetpotato, and affected the overall protein and amino acid content. There was an indirect influence on the accumulation of the metabolites that were associated with amino acid biosynthesis. Nevertheless, the AmA1 expression had no significant effect on the global metabolome in the transgenic tubers. Instead the expression of AmA1 significantly increased the antioxidative activities possibly via the augmentation of phytophenols. The total carbohydrate content was not affected; however, the degradation of starch and cellulose was slower, which might have increased the post-harvest durability of the AmA1-tubers, and would increase the acceptability to the consumers.

## Materials and Methods

### Construction of the *AmA1* transformation vector

The *AmA1* constructs were prepared to effect the expression in sweetpotatoes in constitutive and tuber-specific manner. The plasmid pSB8 was previously constructed using the *Bam*HI*-Eco*RI fragment harbouring the *AmA1* cDNA (912 bp) with 102 bp 3′-UTR region under the control of *CaMV35S* promoter into the binary vector pBI121^18^. For the tuber-specific expression, the CaMV *35S* promoter in pSB8 was replaced by the *Hin*dIII-*Xba*I fragment of sweetpotato *β-amylase*, and the construct was named pSB8β (Fig. 1a,b).

### Genetic transformation and molecular analysis

Sweetpotato cv. SP-6, was obtained from the Central Tuber Crop Research Institute, India and used for the genetic transformations^20^. The integrity of the *AmA1* was assessed by PCR analysis using gene-specific primers (Supplementary Table S4), which delimited the 1.02 kb fragment. The transgene copy number was determined by qRT-PCR using TaqMan chemistry as described earlier^49^ with an ABI PRISM 7700 Sequence Detection System (Applied Biosystems).

### Plant growth, maintenance and sample harvest

The transgenic and WT plants were grown in parallel in greenhouse at identical conditions with one plant per pot (diameter, 30 cm) to eliminate environmental or developmental influence(s). Each line was grown in at least four pots filled with a mix of clay loam and vermiculite (3:1). The mature tubers were harvested, pooled from different plants to normalize the effects of variable growth and development and quickly frozen in liquid nitrogen.

### Immunodetection of AmA1

The tuber proteins were extracted by homogenization in extraction buffer [50 mM Tris-HCl (pH 8.2), 2 mM EDTA, 20% glycerol, 5 mM DTT and 2 mM PMSF]. The homogenates were centrifuged at 10,000 × *g* for 10 min at 4 °C, and the protein concentration in the supernatant was determined using Bradford protein assay kit (Bio-Rad). An aliquot of 50 μg protein from each sample was fractionated on 12.5% (w/v) SDS-PAGE and electrotransferred onto Hybond-C membrane (GE Biosciences). The AmA1 was detected with anti-AmA1 antibody^29^ in combination with alkaline phosphatase conjugated goat anti-rabbit IgG using NBT-BCIP and HRP reaction. The protein quantification was performed with a Fluor-S MultiImager (Bio-Rad) and the band intensity corresponding to AmA1 was quantified using Quantity One software (Bio-Rad).

### Transcript analysis

The transcript accumulation was assessed by northern blot analysis and qRT-PCR. An aliquot of 10 μg of RNA was resolved on 1.2% denaturing agarose gel and transferred onto nylon membrane (GE Healthcare). The hybridization was performed with [^32^P] dCTP labelled *AmA1* and actin cDNA using the random priming kit (New England Biolabs).

The cDNAs for qRT-PCR were prepared using the SuperScript^®^ VILO™ cDNA Synthesis Kit (Invitrogen), and the analysis was performed using gene-specific primers (Supplementary Table S4). The expression data were normalized using actin as an internal control. The qRT-PCR was performed with an ABI PRISM 7700 Sequence Detection System (Applied Biosystems) using SYBR Green dye. The analyses were conducted with two biological and three technical replicates. The mean of Ct values for the target and endogenous reference was used to calculate the relative quantitation (RQ) value using comparative Ct (2^−ΔΔCt^) method.

### Proteome profiling

To examine the effect of AmA1 for the increase in total proteins were isolated from same mass (2 g) of WT and transgenic tubers and aliquots of 100 μl and 250 μl were subjected to 1-DE and 2-DE, respectively. Next, the proteome profiling of the WT and transgenic tubers was accomplished with 250 μg of protein using 2-DE analysis^14^. The electrophoresed proteins were stained with Silver Stain Plus Kit (Bio-Rad). The gel images were scanned by the Fluor-S MultiImager system (Bio-Rad), and analyzed with PDQuest software version 7.2.0 (Bio-Rad). A match set representing a standard image of three replicates, from two biological replicates, was created for each sample. Each spot on the standard gel was quantified with several criteria for consistency in size and shape. The spots with a quality score <30 were eliminated from further analysis.

### Quantitative determination of protein and amino acids

The total protein content of the WT and transgenic tubers was quantified by the micro-Kjeldahl method according to the standard AOAC method^50^. The free amino acids were quantified by the standard ninhydrin method^51^. The amino acids were also quantified with GC-MS using a modified method^52^. The tubers, representing at least four biological replicates, were homogenized in methanol with ribitol as internal standard (2 mg ml^–1^). The extracts were derivatized using methoxyamine hydrochloride (20 mg ml^–1^) in pyridine for 90 min at 30 °C followed by 30 min treatment with MSTFA at 37 °C. Furthermore, the retention time standard mixture (40 μl) was added before trimethylsilylation. The derivatized extracts were diluted in *n*-heptane, and 1 μl sample was injected in splitless mode into the analyzer (Shimadzu GCMS-QP 2010 plus). The GC analyses were performed on an Rtx5MS-30 m column with 0.25 mm ID and df 0.25 (Restek). The injection temperature was set at 260 °C, the interface at 270 °C, and the ion source was adjusted to 230 °C. Helium was used as the carrier gas at a flow rate of 1 ml min^–1^. The mass spectra were recorded at 2 scans s^–1^ with an *m*/*z* 40–600 scanning range. The peaks were assigned and quantified, and the data were normalized to the mean response calculated for the WT. The individual values were normalized against the ribitol. The targeted compounds were analyzed and identified by comparing their retention times and mass spectra with those in the NIST or the Wiley library.

### Assessment of agrophysiological traits

The leaf area of the 3^rd^ to 5^th^ leaf from the shoot apex was measured^53^ and was calculated using LeafJ with ImageJ software (http://rsb.info.nih.gov/ij/). The rate of photosynthesis was quantified with a portable photosynthesis measurement system (GFS3000; Waltz). The photosynthetic potential was determined on the basis of single leaf measurements of 5–7 leaves from each plant and evaluated after 8–10 weeks of growth under standard atmospheric (360 ppm CO_2_) and light conditions (750 μmol m^−2^s^−1^). Identical-sized mature tubers were collected from both the WT and transgenic lines, and the average weight and diameter were measured.

### Analysis of carbohydrates

The total carbohydrates were extracted from the WT and AmA1-tubers, and quantified by the Anthrone method. The values were extrapolated using D-glucose as the standard. The content of reducing sugars was determined by the Nelson-Somogyi method^51^.

To evaluate the degradation of starch and cellulose during storage, the harvested tubers were stored at room temperature and measured at 15-d interval. The total starch content was determined by the Anthrone method with few modifications^51^. The glucose content in the samples was extrapolated using D-glucose as the standard and the starch content was determined by multiplying the values with a factor of 0.9. The total cellulose content was determined by modifying the method of acidolysis with acetic/nitric reagent followed by treatment with H_2_SO_4_. The acid hydrolyzed cellulose was then quantified using the standard procedures^51^.

### Proximate analysis

The proximate analyses were performed according to protocols described previously^14^. Tubers of both the WT and transgenic lines were peeled, sliced into smaller pieces, and oven-dried at 40 °C for 18 h. Total moisture, ash and crude fiber were determined in triplicates according to the standard method^50^.

### Water holding capacity

The WHC was determined using a modified method of Robertson *et al*.^54^. Briefly, 1 g of lyophilized tissue in triplicate was hydrated in 30 ml Milli-Q water containing 0.02% azide. Samples were centrifuged at 3,000 × *g* for 20 min at room temperature after equilibrating them for 18 h. The supernatant was removed and fresh weight was recorded before drying. The WHC was calculated as the amount of water retained by pellet (g/g dry weight) after subtracting the residual dry weight.

### Evaluation of tuber color difference

The high-performance colour measurement spectrophotometer (HunterLab Ultrascan Vis) coupled with EasyMatch QC software was used to determine the colour of tubers. The measurements were performed over 360–780 nm wavelengths, as per manufacturer’s recommendation.

### Determination of phytophenols and carotenoids, and metabolite profiling

The TPC, TFC, TAC and TCC in the tuber extracts were determined by the method described earlier^14^. The metabolite profiling of the WT and two independent transgenic tubers, one each from the COE and TOE lines, was examined by GC-MS analysis.

### Structural characterization of tubers

The **s**tructural analysis of composite samples was conducted using a scanning electron microscope (Model 6390 LV; JOEL Co., Ltd.). The lyophilized samples were coated with 10–15 nm thickness of platinum using a JEOL 1600 Auto Fine Coater. The samples were examined under the SEM with an accelerating voltage of 10–15 KV. The FT-IR spectra were analyzed to determine different functional groups. The compounds were first mixed with approximately 5–7 mg of potassium bromide (KBr) to obtain a fine mixture. The mixture was put in a sample holder and subjected to a hydraulic pressure to produce a KBr compound tablet, which was then transferred in the holder for subsequent characterization.

### Statistical analyses

Statistical significance of data was analyzed by unpaired student’s *t*-test using Graphpad prism 5 software. *P* < *0.05* was considered statistically significant and results were expressed as mean ± s.e.

## Additional Information

**How to cite this article**: Shekhar, S. *et al*. Ectopic expression of amaranth seed storage albumin modulates photoassimilate transport and nutrient acquisition in sweetpotato. *Sci. Rep*. **6**, 25384; doi: 10.1038/srep25384 (2016).

## Supplementary Material

Supplementary Information
