# Supplementary material for: Ectopic expression of amaranth seed storage albumin modulates photoassimilate transport and nutrient acquisition in sweetpotato
Source: Sci Rep. 2016 May 5;6:25384. doi: 10.1038/srep25384 (PMC4857128; doi:10.1038/srep25384)
Supplement: Supplementary Information [file srep25384-s1.pdf]

# Ectopic expression of amaranth seed storage albumin modulates photoassimilate transport and nutrient acquisition in sweetpotato

Shubhendu Shekhar[1,2], Lalit Agrawal[1]*, Divya Mishra[1]*, Alak Kumar Buragohain[2], Mullath Unnikrishnan[3], Chokkappan Mohan[3], Subhra Chakraborty[1§] and Niranjan Chakraborty[1¶]

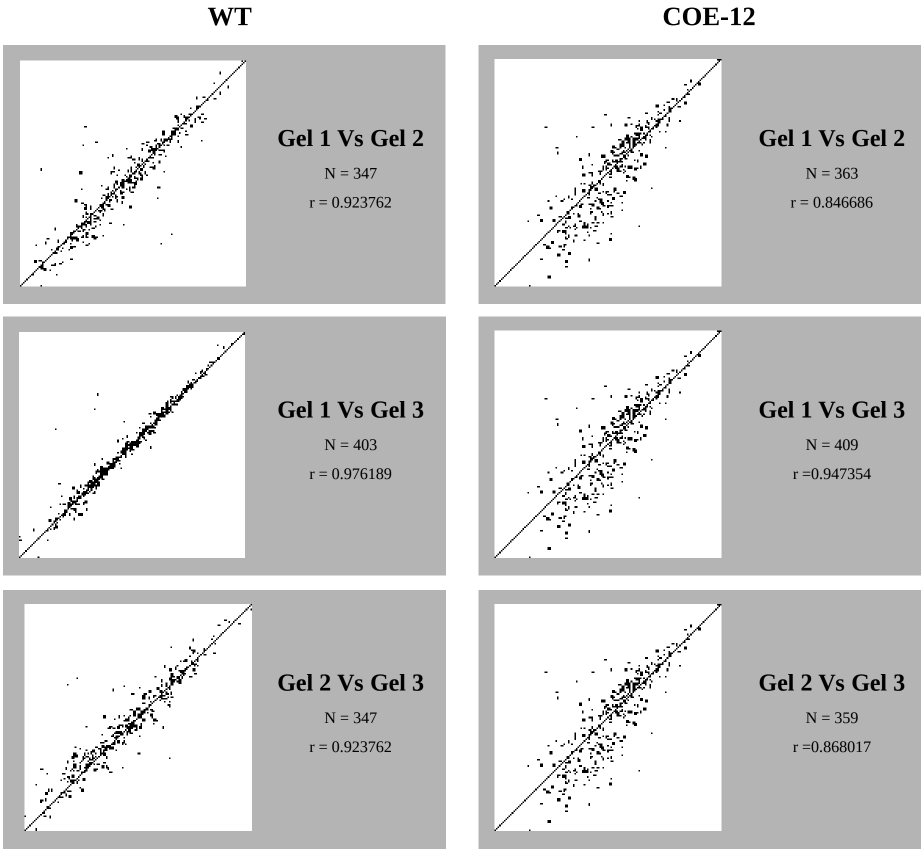


**Supplementary Figure S1. Reproducibility of 2-D gels.** Scatter plots displaying a correlation coefficient of variation above 0.8 among the three replicate gels of WT and COE-12 tuber proteome.

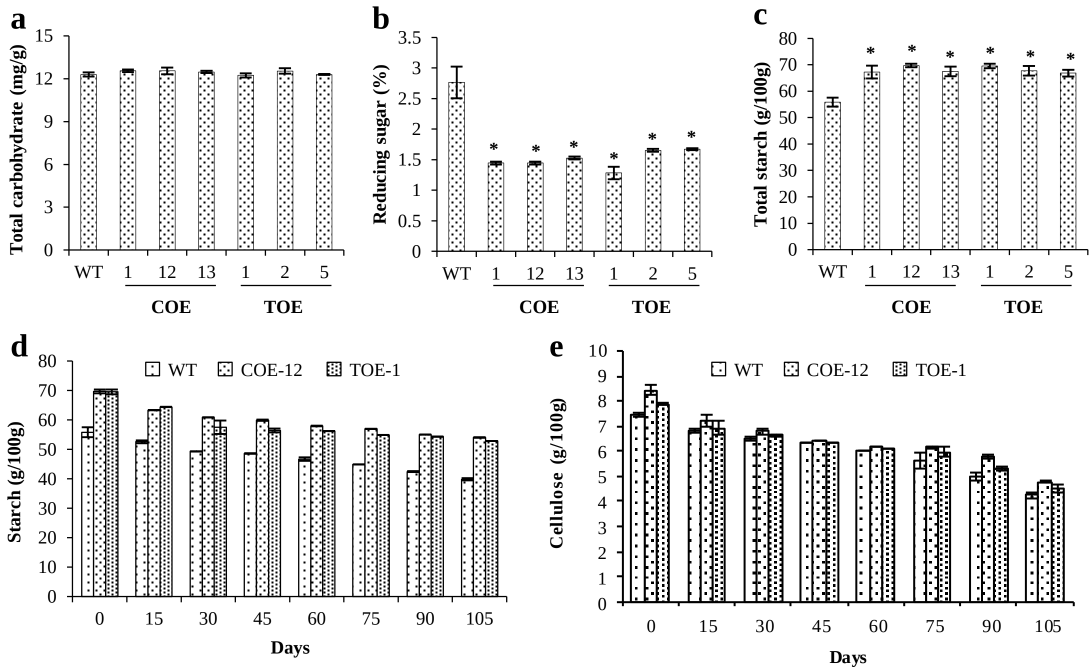


**Supplementary Figure S2. Comparative biochemical analysis.** Total carbohydrate content (**a**), reducing sugar (**b**), and starch content (**c**) of WT and transgenic lines (COE & TOE) were compared. Numerals on X-axis represents the individual lines. Post-harvest stress was evaluated in WT and transgenic tubers. Tubers were stored at room temperature and sampled at every 15-d interval until 105-d. Reduction in starch (**d**) and cellulose (**e**) contents was measured on dry basis at a succession of storage time points. Data represent mean values ± s.e. of three independent measurements and asterisk (*) indicates the level of statistical significance at $p < 0.05$.

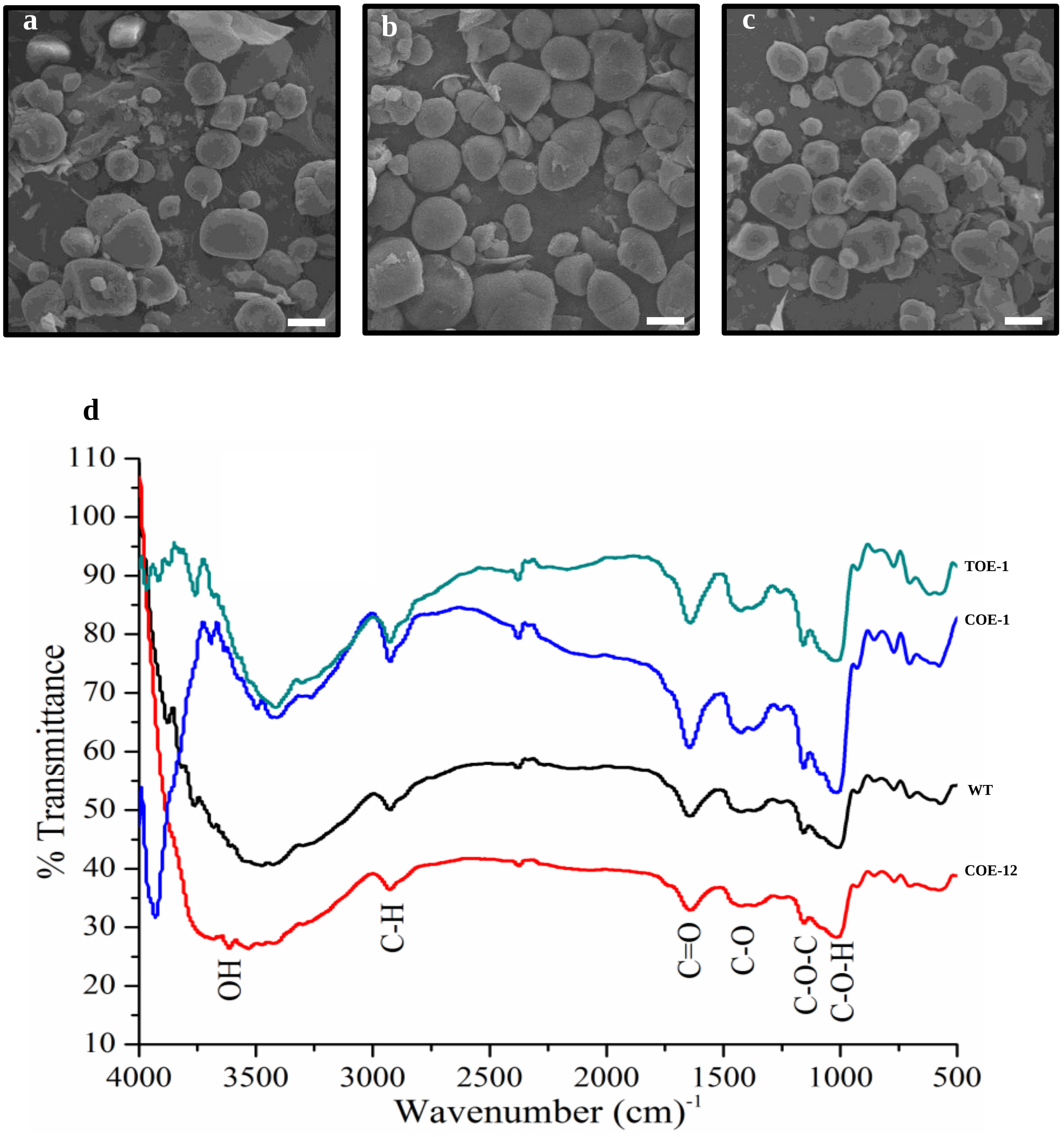


**Supplementary Figure S3. Structural and chemical characterization of tubers.** Structure of starch crystals was visualized by SEM in WT (**a**), COE-12 (**b**) and TOE-1 (**c**) lines. Bar represents 10 µm of magnification. WT and transgenic lines were examined for any possible chemical modifications by FT-IR (**d**).

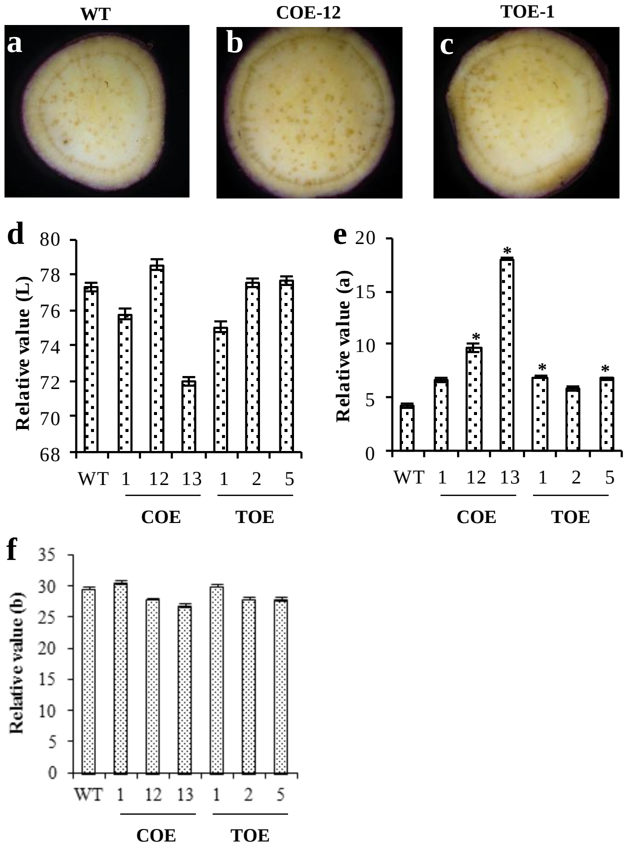


**Supplementary Figure S4. Evaluation of color differences.** Cross sections of tubers displaying the flesh color in WT (**a**), COE-12 (**b**) and TOE-1 (**c**) lines. The relative Hunter 'L' (**d**), 'a' (**e**) and 'b' (**f**) values were determined. Numbers on X-axis represents individual lines. Data represent mean values ± s.e. (n=3) and asterisk (*) indicates the level of statistical significance at $p < 0.05$.

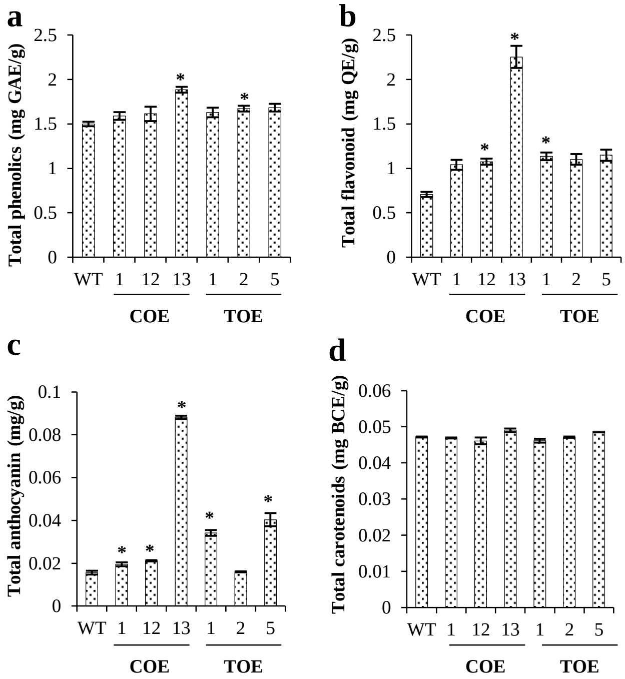


**Supplementary Figure S5. Determination of phytophenols and carotenoids.** A comparative analysis of TPC (**a**), TFC (**b**), TAC (**c**) and TCC (**d**) determined on dry basis in WT and transgenic (COE and TOE) lines. Values are presented as means ± s.e. (n=3) of a composite sample of four to eight tubers. Numbers on X-axis represents individual lines. Asterisks (*) indicate the level of significance at $p < 0.05$. GAE: gallic acid equivalent; QE: quercetin equivalent; BCE: β-carotene equivalents.

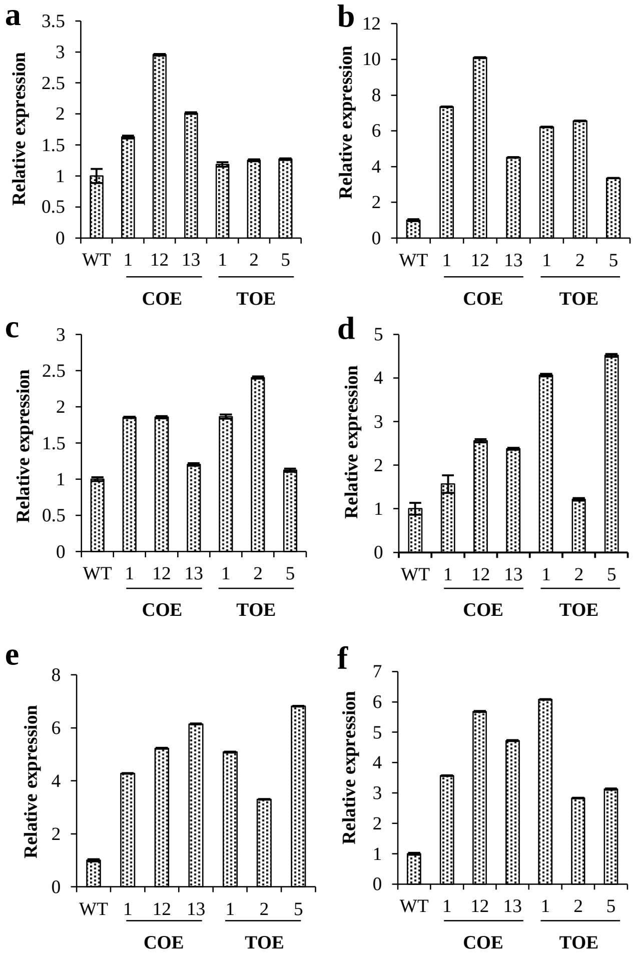


**Supplementary Figure S6. Transcript abundance of flavonoid pathway genes.** Relative abundance of chalcone synthase (*CHS*) (**a**), chalcone flavanone isomerase (*CHI*) (**b**), flavanone 3-hydroxylase (*F3H*) (**c**), dihydroflavonol 4-reductase (*DFR*) (**d**), anthocyanidin synthase (*ANS*) (**e**) and UDP-glucose flavonoid 3-O-glucosyl transferase (*UFGT*) (**F**) was compared in WT and transgenic lines. Numbers on X-axis represents individual lines. The mean values of three replicates were normalized using actin as internal control. Values represent mean ± s.e.

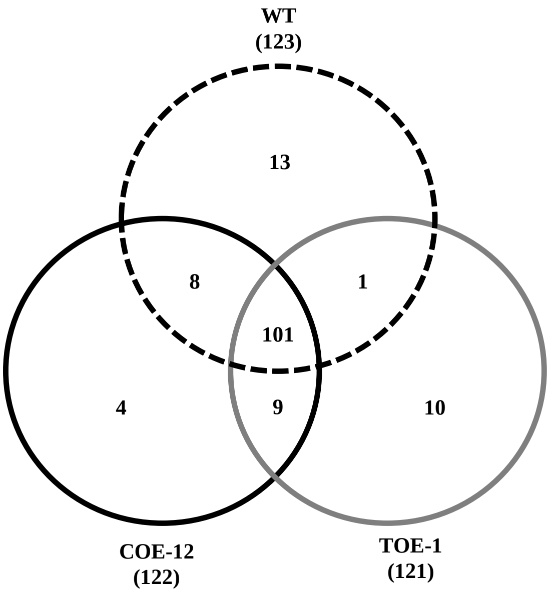


**Supplementary Figure S7. Allocation of metabolites in WT and transgenic events.** Common and exclusive metabolites across WT and transgenic lines (COE-12 and TOE-1) are shown in Venn diagram. The areas in the diagram are not proportional to the number of metabolites in the groups.

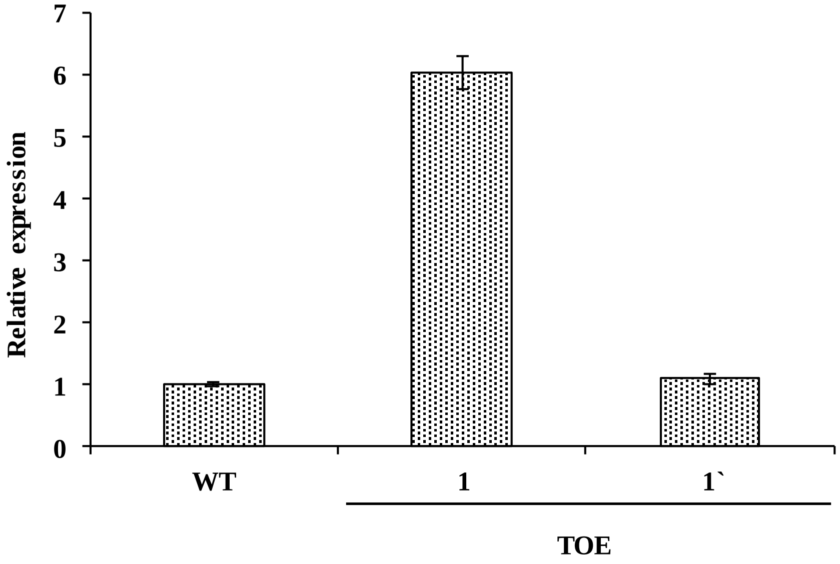


**Supplementary Figure S8. Analysis of leaky expression in TOE line.** Leaky expression was checked in aerial parts of TOE lines. Numerical 1 and 1' represent tubers and aerial parts, respectively.

**Supplementary Table 1: Reproducibility of 2-D gels**

| Lines | Average no of spots | High quality spots | Reproducibility (%) |
|---|---|---|---|
| WT | 376 | 367 | 97.61 |
| COE-12 | 384 | 370 | 96.35 |

**Supplementary Table S2: Proximate composition and water holding capacity**

| Lines | Moisture (%) | Ash (%) | Fiber (%) | WHC ($g\ g^{-1}$) |
|---|---|---|---|---|
| WT | 71.42 ± 0.33 | 4.7 ± 0.05 | 2.35 ± 0.15 | 1.43 ± 0.13 |
| COE | | | | |
| 1 | 72.34 ± 0.42 | 4.67 ± 0.37 | 2.34 ± 0.04 | 1.49 ± 0.11 |
| 12 | 73.88 ± 0.64 | 4.73 ± 0.05 | 2.36 ± 0.10 | 1.47 ± 0.02 |
| 13 | 73.77 ± 0.22 | 4.76 ± 0.11 | 2.35 ± 0.01 | 2.06 ± 0.22* |
| TOE | | | | |
| 1 | 72.28 ± 0.36 | 4.83 ± 0.28 | 2.33 ± 0.03 | 1.45 ± 0.03 |
| 2 | 72.33 ± 0.26 | 4.77 ± 0.40 | 2.34 ± 0.03 | 1.53 ± 0.04 |
| 5 | 72.75 ± 0.09 | 4.73 ± 0.50 | 2.35 ± 0.04 | 1.48 ± 0.11 |

Values are presented as means ± SE (n=3) of a composite sample of four to eight tubers. * indicates the level of significance at $p < 0.05$.

Supplementary Table S3: Comparative metabolome profiling and allocation of metabolites

**WT**

| Name | RT |
|---|---|
| Glycine, N,N-bis(trimethylsilyl)-, trimethylsilyl ester | 6.471 |
| Butanedioic acid, bis(trimethylsilyl) ester | 6.565 |
| L-Leucine-2TMS | 6.63 |
| Propanoic acid, 2,3-bis[(trimethylsilyl)oxy]-, trimethylsilyl ester | 6.886 |
| 2-Butenedioic acid (E)-, bis(trimethylsilyl) ester | 7.031 |
| Nonanoic acid, trimethylsilyl ester | 7.183 |
| Serine tritms | 7.305 |
| N,O,O-Tris(trimethylsilyl)-L-threonine | 7.688 |
| l-Aspartic acid, bis(trimethylsilyl) ester | 8.142 |
| D,L-Alanine, N-(tert-butyldimethylsilyl)-N-methyl-, tert-butyldimethylsilyl ester | 8.347 |
| Valylvaline, N,N'-dimethyl-n-propoxycarbonyl-, butyl ester | 8.351 |
| SILANOL, TRIMETHYL-, PHOSPHATE | 8.808 |
| Malic acid, O-(trimethylsilyl)-, bis(trimethylsilyl)ester | 9.058 |
| 2,4(1H,3H)-Pyrimidinedione, dihydro-1,3-bis(trimethylsilyl)- | 9.19 |
| BUTANAL, 2,3,4-TRIS[(TRIMETHYLSILYL)OXY]- | 9.366 |
| L-ASPARTIC ACID, N-(TRIMETHYLSILYL)- | 9.457 |
| Butanoic acid, 4-[bis(trimethylsilyl)amino]-, trimethylsilyl ester | 9.515 |
| Undecanoic acid, trimethylsilyl ester | 9.748 |
| Phenylalanine-2TMS | 9.81 |
| Propanoic acid, 3-[bis(trimethylsilyl)amino]-2-methyl-, trimethylsilyl ester | 9.915 |
| L-Threonic acid, tris(trimethylsilyl) ether, trimethylsilyl ester | 10.038 |
| L-Asparagine, N2-trimethylsilyl-, trimethylsilyl ester | 10.417 |
| D,L-Alanine, N-(tert-butyldimethylsilyl)-N-methyl-, tert-butyldimethylsilyl ester | 10.561 |
| Glutamine, tris(trimethylsilyl)- | 10.651 |
| 2,3,4,5-Tetrahydroxypentanoic acid-1,4-lactone, tris(trimethylsilyl)- | 10.776 |
| 2,3,4,5-Tetrahydroxypentanoic acid-1,4-lactone | 10.921 |
| Gluconic acid, 2-methoxime, tetra(trimethylsilyl)-, trimethylsilyl ester | 11.128 |
| L-Asparagine, N,N2-bis(trimethylsilyl)-, trimethylsilyl ester | 11.293 |
| 1,2-Epoxy-3,4-dihydroxycyclohexano[a]pyrene | 11.36 |
| d-Ribose, 2,3,4,5-tetrakis-O-(trimethylsilyl)-, O-methyloxime | 11.454 |
| ARABINONIC ACID, 2,3,5-TRIS-O-(TRIMETHYLSILYL)-, .GAMMA.-LACTONE | 11.541 |
| Xylitol, 1,2,3,4,5-pentakis-O-(trimethylsilyl) | 11.643 |
| 1-Dodecanol, 3,7,11-trimethyl | 11.751 |
| Ribitol, 1,2,3,4,5-pentakis-O-(trimethylsilyl)- | 11.776 |
| L-Lysine-4TMS | 11.88 |
| l-(-)-Arabitol, pentakis(trimethylsilyl) ether | 11.976 |
| Xylonic acid, 2,3,5-tris-O-(trimethylsilyl) | 12.08 |
| D-GLUCITOL, 1,2,3,4,5,6-HEXAKIS-O-(TRIMETHYLSILYL) | 12.154 |
| Ribonic acid, 2,3,4,5-tetrakis-O-(trimethylsilyl)-, trimethylsilyl ester | 12.282 |
| Undecanoic acid, tert-butyldimethylsilyl ester | 12.326 |
| Ribonic acid, 2,3,4,5-tetrakis-O-(trimethylsilyl)-, trimethylsilyl ester | 12.428 |
| Ribonic acid, 2,3,4,5-tetrakis-O-(trimethylsilyl)-, trimethylsilyl ester | 12.522 |
| Azelaic acid, bis(trimethylsilyl) ester | 12.575 |
| ({4,5-BIS[(TRIMETHYLSILYL)OXY]TETRAHYDRO-3-FURANYL}OXY)(TRIMETHYL)SILANE | 12.783 |
| L-Valine, N-(trimethylsilyl)-, trimethylsilyl ester | 12.858 |
| [1,1'-BIPHENYL]-4-CARBOXYLIC ACID, TRIMETHYLSILYL ESTER | 12.953 |
| Tetradecanoic acid, trimethylsilyl ester | 13.064 |
| D-Fructose, 1,3,4,5,6-pentakis-O-(trimethylsilyl)-, O-methyloxime | 13.211 |
| 2-Thiobarbituric acid, S-trimethylsilyl-, bis(trimethylsilyl) ether | 13.424 |
| D-Fructose, 1,3,4,5,6-pentakis-O-(trimethylsilyl)-, O-methyloxime | 13.576 |
| D-Fructose, 1,3,4,5,6-pentakis-O-(trimethylsilyl)-, O-methyloxime | 13.683 |
| d-Mannose, 2,3,4,5,6-pentakis-O-(trimethylsilyl)-, o-methyloxyme, (1Z) | 13.767 |
| GALACTOSE OXIME 6TMS | 13.836 |
| D-GLUCITOL, 1,2,3,4,5,6-HEXAKIS-O-(TRIMETHYLSILYL) | 14.018 |
| (1E,3Z)-1-PHENYL-3-(TRIMETHYLSILYLOXY)-1,3-PENTADIENE | 14.114 |
| D-GLUCITOL, 1,2,3,4,5,6-HEXAKIS-O-(TRIMETHYLSILYL) | 14.13 |
| Gulonic acid, 2,3,5,6-tetrakis-O-(trimethylsilyl)-, lactone | 14.232 |
| cis-9-Hexadecenoic acid, trimethylsilyl ester | 14.818 |
| 1,2,3-PROPANETRICARBOXYLIC ACID, 2-[(TRIMETHYLSILYL)OXY]- | 14.858 |
| Hexadecanoic acid, trimethylsilyl ester | 15.005 |
| GLUCARIC ACID, 2,3,4,5-TETRAKIS-O-(TRIMETHYLSILYL)-, BIS(TRIMETHYLSILYL) ESTER | 15.27 |
| Tetradecanoic acid, dimethyl(isopropyl)silyl ester | 15.314 |
| Heptadecanoic acid, trimethylsilyl ester | 15.573 |
| Myo-Inositol, 1,2,3,4,5,6-hexakis-O-(trimethylsilyl)- | 15.67 |
| alpha.-D-Glucopyranoside, methyl 2,3-bis-O-(trimethylsilyl)-, cyclic methylboronate | 15.767 |
| TRIMETHYL({2,3,4,5,6-PENTAKIS[(TRIMETHYLSILYL)OXY]CYCLOHEXYL}OXY)SILANE | 15.826 |
| Heptadecanoic acid, trimethylsilyl ester | 15.908 |
| 2,4'-Bis(trimethylsilyloxy)diphenylmethane | 16.111 |
| Silane, dimethylbis[(trimethylsilyl)methyl] | 16.2 |
| OELSAEURE, TRIMETHYLSILYLESTER | 16.566 |
| OLEIC ACID, TRIMETHYLSILYL ESTER | 16.626 |
| Octadecanoic acid, trimethylsilyl ester | 16.783 |
| 9,12-Octadecadienoic acid (Z,Z)-, trimethylsilyl ester | 16.948 |
| Hexadecanoic acid, tert-butyldimethylsilyl ester | 17.111 |
| 9,12-Octadecadienoic acid (Z,Z)-, trimethylsilyl ester | 17.19 |
| 9,12-Octadecadienoic acid (Z,Z)-, trimethylsilyl ester | 17.322 |
| cis-15-Tetracosenoic acid, trimethylsilyl ester | 17.533 |
| Nonadecanoic acid, trimethylsilyl ester | 17.619 |

| Name | RT |
|---|---|
| Ferulic acid, trimethylsiloxy, trimethylsilyl ester | 17.78 |
| 1,6,10,14,18,22-Tetracosahexaen-3-ol, 2,6,10,15,19,23-hexamethyl | 17.917 |
| 1,5,9-Decatriene, 2,3,5,8-tetramethyl- | 17.925 |
| Heptadecanoic acid, tert-butyldimethylsilyl ester | 17.955 |
| Ricinoleic acid, trimethylsiloxy, trimethylsilyl ester | 18.221 |
| cis-13-Eicosenoic acid, trimethylsilyl ester | 18.3 |
| Eicosanoic acid, trimethylsilyl ester | 18.444 |
| Myo-Inositol, 1,3,4,5,6-pentakis-O-(trimethylsilyl)-, bis(trimethylsilyl) phosphate | 18.578 |
| 17-Octadecynoic acid, tert-butyldimethylsilyl ester | 18.582 |
| trans-9-Octadecenoic acid, tert-butyldimethylsilyl ester | 18.672 |
| Uridine, 2',3',5'-tris-O-(trimethylsilyl)- | 18.804 |
| Octadecanoic acid, tert-butyldimethylsilyl ester | 18.827 |
| 9,12-Octadecadienoic acid (Z,Z)-, trimethylsilyl ester | 19.049 |
| Heneicosanoic acid, trimethylsilyl ester | 19.39 |
| 9,12-Octadecadienoic acid, tert-butyldimethylsilyl ester, (Z,Z)- | 19.485 |
| alpha.-D-Glucopyranoside, 1,3,4,6-tetrakis-O-(trimethylsilyl)-.beta.-D-fructofuranosyl 2,3,4,6-tetrakis-O-(trimethylsilyl) | 19.7 |
| Nonadecanoic acid, tert-butyldimethylsilyl ester | 19.871 |
| Hexadecanoic acid, 2,3-bis[(trimethylsilyl)oxy]propyl ester | 20.034 |
| Per-O-trimethylsilyl-(3-O-.alpha.-d-mannopyranosyl-4-O-.beta.-d-glucopyranosyl-d-glucitol) | 20.265 |
| Docosanoic acid, trimethylsilyl ester | 20.544 |
| Ricinoleic acid, trimethylsiloxy, trimethylsilyl ester | 20.725 |
| 10-Undecenoic acid, tert-butyldimethylsilyl ester | 20.797 |
| D-Turanose, heptakis(trimethylsilyl)- | 21.002 |
| alpha.-D-Glucopyranoside, 1,3,4,6-tetrakis-O-(trimethylsilyl)-.beta.-D-fructofuranosyl 2,3,4,6-tetrakis-O-(trimethylsilyl)- | 21.362 |
| Tetracosanoic acid, trimethylsilyl ester | 21.969 |
| D-Turanose, heptakis(trimethylsilyl)- | 22.16 |
| D-Turanose, heptakis(trimethylsilyl)- | 22.351 |
| L-Methionine-2TMS | 22.611 |
| D-Fructose, 3-O-[2,3,4,6-tetrakis-O-(trimethylsilyl)-.alpha.-D-glucopyranosyl]-1,4,5,6-tetrakis-O-(trimethylsilyl)- | 22.988 |
| D-Turanose, heptakis(trimethylsilyl)- | 23.306 |
| D-Turanose, heptakis(trimethylsilyl)- | 23.511 |
| Tetracosanoic acid, trimethylsilyl ester | 23.77 |
| Docosanoic acid, tert-butyldimethylsilyl ester | 24.795 |
| Cholest-5-en-3-ol (3.beta.)-, carbonochloridate | 25.284 |
| Ricinoleic acid, trimethylsiloxy, trimethylsilyl ester | 26.11 |
| Tricosanoic acid, tert-butyldimethylsilyl ester | 26.592 |
| Tocopherol-.beta.-tms-derivative | 26.883 |
| Hexacosanoic acid, trimethylsilyl ester | 27.217 |
| Cholesta-2,4-diene | 27.242 |
| Melibiose, octakis(trimethylsilyl) | 27.352 |
| STIGMAST-5-EN-3-OL, (3.BETA.) | 27.546 |
| D-Glucose, 4-O-[2,3,4,6-tetrakis-O-(trimethylsilyl)-.beta.-D-galactopyranosyl]-2,3,5,6-tetrakis-O-(trimethylsilyl)- | 27.627 |
| Tetracosanoic acid, tert-butyldimethylsilyl ester | 27.727 |
| Stigmast-5-en-3-ol, oleate | 27.893 |
| 9,19-Cyclolanost-23-ene-3,25-diol, 3-acetate, (3.beta.,23E) | 28.065 |
| Hexacosanoic acid, trimethylsilyl ester | 28.166 |
| alpha.-Tocopherol (vitamin E), trimethysilyl derivative | 28.313 |
| SILANE, [[(3.BETA.)-CHOLEST-5-EN-3-YL]OXY]TRIMETHYL- | 28.4 |
| 4.alpha.,5-Cyclo-A-homo-5.alpha.-cholestan-6-one | 28.55 |
| Pentacosanoic acid, tert-butyldimethylsilyl ester | 28.577 |
| GLUCOPYRANOSIDE-6,6-D2, METHYL-TETRAKIS-O-(TRIMETHYLSILYL)- | 28.726 |
| THREONIC ACID, 2,3-BIS-O-(TRIMETHYLSILYL)-, .GAMMA.-LACTONE, D- | 28.7 |
| Silane, trimethyl[(3,7,11-trimethyl-2,6,10-dodecatrienyl)oxy]- | 28.876 |
| SOLANESOL | 28.881 |
| 10,12-Tricosadiynoic acid, trimethylsilyl ester | 29.1 |
| Campesterol tms | 29.218 |
| TETRAHYDRODAMMARADIENOL | 29.231 |
| Stigmasterol trimethylsilyl ether | 29.438 |
| XYLOPYRANOSIDE, METHYL 2,3,4-TRIS-O-(TRIMETHYLSILYL)-, .ALPHA.-D- | 29.667 |
| STIGMAST-5-EN-3-OL, (3.BETA.)- | 29.75 |
| Betulin | 29.767 |
| beta.-Sitosterol trimethylsilyl ether | 29.88 |
| Silane, (1,1-dimethylethyl)dimethyl(octacosyloxy) | 30.048 |
| 1,2,2,3,4,4,5,5,6,6-Decamethylhexasilinane-1,3-diol | 30.266 |
| Hexopyranose, 1,2,3,4,6-pentakis-O-(trimethylsilyl) | 30.401 |
| Ergostane-5,25-diol, 3,6,12-tris[(trimethylsilyl)oxy]-, 25-acetate, (3.beta.,5.alpha.,6.beta.,12.beta.)- | 30.404 |
| alpha.-D-Glucopyranoside, 1,3,4,6-tetrakis-O-(trimethylsilyl)-.beta.-D-fructofuranosyl 2,3,4,6-tetrakis-O-(trimethylsilyl)- | 30.688 |
| alpha.-D-Glucopyranoside, 1,3,4,6-tetrakis-O-(trimethylsilyl)-.beta.-D-fructofuranosyl 2,3,4,6-tetrakis-O-(trimethylsilyl)- | 30.791 |
| TMS ETHER OF 2-MONOOLEGLYCEROL | 31.03 |
| Silane, (9,19-cyclo-9.beta.-lanost-24-en-3.beta.-yloxy)trimethyl- | 31.61 |
| 14,17-Nor-3,21-dioxo-.beta.-amyrin, 17,18-didehydro-3-dehydroxy- | 31.725 |
| beta.-D-Glucopyranose, 2,3,4,6-tetrakis-O-(trimethylsilyl)-, 1-(trimethylsilyl)-1H-indole-3-acetate | 31.895 |
| METHYL 3-TRIMETHYLSILYLOXYTETRADECANOATE | 32.05 |
| SOLANESOL | 32.361 |
| Olean-12-en-28-oic acid, 3-(acetyloxy)-, methyl ester, (3.beta.) | 32.996 |

**COE-12**

| Name | RT |
|---|---|
| L-Leucine-2TMS | 6.63 |
| PROPANOIC ACID, 2,3-BIS[(TRIMETHYLSILYL)OXY]-, TRIMETHYLSILYL ESTER | 6.89 |
| 1-Butanol, 2-methyl-, acetate | 7 |
| 1,2-Ethanediol, monoacetate | 7.008 |
| 2-Butenedioic acid (Z)-, bis(trimethylsilyl) ester | 7.033 |
| Nonanoic acid, trimethylsilyl ester | 7.186 |
| Serine tritms | 7.305 |
| N,O,O-Tris(trimethylsilyl)-L-threonine | 7.687 |
| l-Aspartic acid, bis(trimethylsilyl) ester | 8.144 |
| l-Norvaline, n-butoxycarbonyl-, butyl ester | 8.348 |
| Valylvaline, N,N'-dimethyl-n-propoxycarbonyl-, butyl ester | 8.351 |
| SILANOL, TRIMETHYL-, PHOSPHATE | 8.816 |
| Malic acid, O-(trimethylsilyl)-, bis(trimethylsilyl)ester | 9.059 |
| 2,4(1H,3H)-Pyrimidinedione, dihydro-1,3-bis(trimethylsilyl)- | 9.19 |
| BUTANAL, 2,3,4-TRIS[(TRIMETHYLSILYL)OXY]-, (R*,R*)- | 9.367 |
| L-ASPARTIC ACID, N-(TRIMETHYLSILYL)-, BIS(TRIMETHYLSILYL) ESTER | 9.457 |
| Butanoic acid, 4-[bis(trimethylsilyl)amino]-, trimethylsilyl ester | 9.515 |
| Undecanoic acid, trimethylsilyl ester | 9.749 |
| Phenylalanine-2TMS | 9.81 |
| Propanoic acid, 2-methyl-2,3-bis[(trimethylsilyl)oxy]-, trimethylsilyl ester | 9.915 |
| L-Threonic acid, tris(trimethylsilyl) ether, trimethylsilyl ester | 10.037 |
| L-Asparagine, N2-trimethylsilyl-, trimethylsilyl ester | 10.407 |
| D,L-Alanine, N-(tert-butyldimethylsilyl)-N-methyl-, tert-butyldimethylsilyl ester | 10.561 |
| Glutamine, tris(trimethylsilyl) | 10.65 |
| PENTITOL, 3-DESOXY-TETRAKIS-O-(TRIMETHYLSILYL)- | 10.776 |
| 3,8-Dioxa-2,9-disiladecane, 2,2,9,9-tetramethyl-5,6-bis[(trimethylsilyl)oxy] | 10.919 |
| Pentonic acid, 3-deoxy-2,4,5-tris-O-(trimethylsilyl)-, trimethylsilyl ester | 11.058 |
| Gluconic acid, 2-methoxime, tetra(trimethylsilyl)-, trimethylsilyl ester | 11.129 |
| L-Asparagine, N,N2-bis(trimethylsilyl)-, trimethylsilyl ester | 11.293 |
| 1,2-Epoxy-3,4-dihydroxycyclohexano[a]pyrene | 11.35 |
| Butanedioic acid, [(trimethylsilyl)oxy]-, bis(trimethylsilyl) ester | 11.417 |
| d-Ribose, 2,3,4,5-tetrakis-O-(trimethylsilyl)-, O-methyloxime | 11.454 |
| D-Arabinonic acid, 2,3,5-tris-O-(trimethylsilyl)-, .gamma.-lactone | 11.533 |
| Xylitol, 1,2,3,4,5-pentakis-O-(trimethylsilyl) | 11.643 |
| 1-Dodecanol, 3,7,11-trimethyl | 11.754 |
| Ribitol, 1,2,3,4,5-pentakis-O-(trimethylsilyl)- | 11.776 |
| L-Lysine-4TMS | 11.88 |
| l-(-)-Arabitol, pentakis(trimethylsilyl) ether | 11.976 |
| Xylonic acid, 2,3,5-tris-O-(trimethylsilyl)-, .gamma.-lactone, D- | 12.067 |
| D-GLUCITOL, 1,2,3,4,5,6-HEXAKIS-O-(TRIMETHYLSILYL) | 12.154 |
| Ribonic acid, 2,3,4,5-tetrakis-O-(trimethylsilyl)-, trimethylsilyl ester | 12.282 |
| Undecanoic acid, tert-butyldimethylsilyl ester | 12.326 |
| Ribonic acid, 2,3,4,5-tetrakis-O-(trimethylsilyl)-, trimethylsilyl ester | 12.425 |
| Ribonic acid, 2,3,4,5-tetrakis-O-(trimethylsilyl)-, trimethylsilyl ester | 12.523 |
| Azelaic acid, bis(trimethylsilyl) ester | 12.575 |
| MANNOFURANOSIDE, METHYL 2,3,5,6-TETRAKIS-O-(TRIMETHYLSILYL)-, .ALPHA.-D- | 12.758 |
| L-Valine, N-(trimethylsilyl)-, trimethylsilyl ester | 12.86 |
| 1,1'-BIPHENYL]-4-CARBOXYLIC ACID, TRIMETHYLSILYL ESTER | 12.956 |
| Tetradecanoic acid, trimethylsilyl ester | 13.063 |
| D-Fructose, 1,3,4,5,6-pentakis-O-(trimethylsilyl)-, O-methyloxime | 13.214 |
| 2,3,4,6-Tetra-O-trimethylsilyl-1-[1,7-dicarba-closo-dodecaboran-(12)-1-yl]-D-glucopyranose | 13.254 |
| 2-Thiobarbituric acid, S-trimethylsilyl-, bis(trimethylsilyl) ether | 13.425 |
| D-FRUCTOSE, O-METHYLOXIM, PENTAKIS-O-(TRIMETHYLSILYL)- | 13.577 |
| D-Fructose, 1,3,4,5,6-pentakis-O-(trimethylsilyl)-, O-methyloxime | 13.683 |
| GALACTOSE OXIME 6TMS | 13.836 |
| D-GLUCITOL, 1,2,3,4,5,6-HEXAKIS-O-(TRIMETHYLSILYL)- | 14.018 |
| D-Glycero-L-manno-Heptonic acid, 2,3,5,6,7-pentakis-O-(trimethylsilyl) | 14.122 |
| Gulonic acid, 2,3,5,6-tetrakis-O-(trimethylsilyl)-, lactone | 14.235 |
| L-ASPARTIC ACID, N-(TRIMETHYLSILYL)-, BIS(TRIMETHYLSILYL) ESTER | 14.312 |
| D-Galactose, 2,3,4,5,6-pentakis-O-(trimethylsilyl) | 14.661 |
| PALMITELAIDIC ACID 1TMS | 14.817 |
| 1,2,3-PROPANETRICARBOXYLIC ACID, 2-[(TRIMETHYLSILYL)OXY]-, TRIS(TRIMETHYLSILYL) ESTER | 14.858 |
| Hexadecanoic acid, trimethylsilyl ester | 15.005 |
| 1,2,3,4,6-PENTAKIS-O-(TRIMETHYLSILYL)HEXOPYRANOSE | 15.13 |
| GLUCARIC ACID, 2,3,4,5-TETRAKIS-O-(TRIMETHYLSILYL)-, BIS(TRIMETHYLSILYL) ESTER | 15.271 |
| Tetradecanoic acid, dimethyl(isopropyl)silyl ester | 15.315 |
| Gulonic acid, 2,3,5,6-tetrakis-O-(trimethylsilyl)-, lactone | 15.368 |
| Heptadecanoic acid, trimethylsilyl ester | 15.572 |
| Myo-Inositol, 1,2,3,4,5,6-hexakis-O-(trimethylsilyl)- | 15.671 |
| TRIMETHYL({2,3,4,5,6-PENTAKIS[(TRIMETHYLSILYL)OXY]CYCLOHEXYL}OXY)SILANE | 15.673 |
| TRIMETHYL({2,3,4,5,6-PENTAKIS[(TRIMETHYLSILYL)OXY]CYCLOHEXYL}OXY)SILANE | 15.826 |
| 2,4'-Bis(trimethylsilyloxy)diphenylmethane | 15.827 |
| Heptadecanoic acid, trimethylsilyl ester | 15.904 |
| 2,4'-Bis(trimethylsilyloxy)diphenylmethane | 16.113 |
| OELSAEURE, TRIMETHYLSILYLESTER | 16.565 |
| OLEIC ACID, TRIMETHYLSILYL ESTER | 16.625 |
| Octadecanoic acid, trimethylsilyl ester | 16.782 |
| 9,12-Octadecadienoic acid (Z,Z)-, trimethylsilyl ester | 16.946 |

| Name | RT |
|---|---|
| Hexadecanoic acid, tert-butyldimethylsilyl ester | 17.107 |
| 9,12-Octadecadienoic acid (Z,Z)-, trimethylsilyl ester | 17.324 |
| Nonadecanoic acid, trimethylsilyl ester | 17.619 |
| Ferulic acid, trimethylsiloxy, trimethylsilyl ester | 17.783 |
| Isopimaric acid TMS | 17.907 |
| 1,5,9-DECATRIEN, 2,3,5,8-TETRAMETHYL | 17.925 |
| 9-Octadecenoic acid, 2-[(trimethylsilyl)oxy]-1-[[(trimethylsilyl)oxy]methyl]ethyl ester | 17.945 |
| Heptadecanoic acid, tert-butyldimethylsilyl ester | 17.952 |
| Ricinoleic acid, trimethylsiloxy, trimethylsilyl ester | 18.223 |
| cis-13-Eicosenoic acid, trimethylsilyl ester | 18.3 |
| Eicosanoic acid, trimethylsilyl ester | 18.443 |
| D-Myo-Inositol, 1,2,4,5,6-pentakis-O-(trimethylsilyl)-, bis(trimethylsilyl) phosphate | 18.576 |
| 17-Octadecynoic acid, tert-butyldimethylsilyl ester | 18.582 |
| trans-9-Octadecenoic acid, tert-butyldimethylsilyl ester | 18.672 |
| Octadecanoic acid, tert-butyldimethylsilyl ester | 18.813 |
| ANDROSTANE, SILANE DERIV. | 19.033 |
| Heneicosanoic acid, trimethylsilyl ester | 19.389 |
| 9,12-Octadecadienoic acid, tert-butyldimethylsilyl ester, (Z,Z)- | 19.487 |
| alpha.-D-Glucopyranoside, 1,3,4,6-tetrakis-O-(trimethylsilyl)-.beta.-D-fructofuranosyl 2,3,4,6-tetrakis-O-(trimethylsilyl) | 19.697 |
| Nonadecanoic acid, tert-butyldimethylsilyl ester | 19.873 |
| Hexadecanoic acid, 2,3-bis[(trimethylsilyl)oxy]propyl ester | 20.033 |
| Per-O-trimethylsilyl-(3-O-.alpha.-d-mannopyranosyl-4-O-.beta.-d-glucopyranosyl-d-glucitol) | 20.267 |
| Docosanoic acid, trimethylsilyl ester | 20.543 |
| Ricinoleic acid, trimethylsiloxy, trimethylsilyl ester | 20.727 |
| D-Turanose, heptakis(trimethylsilyl) | 21.011 |
| alpha.-D-Glucopyranoside, 1,3,4,6-tetrakis-O-(trimethylsilyl)-.beta.-D-fructofuranosyl 2,3,4,6-tetrakis-O-(trimethylsilyl) | 21.388 |
| Tetracosanoic acid, trimethylsilyl ester | 21.968 |
| D-Turanose, heptakis(trimethylsilyl) | 22.161 |
| D-Fructose, 3-O-[2,3,4,6-tetrakis-O-(trimethylsilyl)-.alpha.-D-glucopyranosyl]-1,4,5,6-tetrakis-O-(trimethylsilyl) | 22.35 |
| L-Methionine-2TMS | 22.611 |
| D-Fructose, 3-O-[2,3,4,6-tetrakis-O-(trimethylsilyl)-.alpha.-D-glucopyranosyl]-1,4,5,6-tetrakis-O-(trimethylsilyl)- | 22.987 |
| D-Turanose, heptakis(trimethylsilyl) | 23.307 |
| D-Turanose, heptakis(trimethylsilyl)- | 23.509 |
| Tetracosanoic acid, trimethylsilyl ester | 23.764 |
| Docosanoic acid, tert-butyldimethylsilyl ester | 24.796 |
| Cholest-5-en-3-ol (3.beta.)-, carbonochloridate | 25.287 |
| Tricosanoic acid, tert-butyldimethylsilyl ester | 26.602 |
| Ricinoleic acid, trimethylsiloxy, trimethylsilyl ester | 26.112 |
| Tocopherol-.beta.-tms-derivative | 26.884 |
| Hexacosanoic acid, trimethylsilyl ester | 27.208 |
| Melibiose, octakis(trimethylsilyl) | 27.352 |
| STIGMAST-5-EN-3-OL, (3.BETA.) | 27.546 |
| D-Glucose, 4-O-[2,3,4,6-tetrakis-O-(trimethylsilyl)-.beta.-D-galactopyranosyl]-2,3,5,6-tetrakis-O-(trimethylsilyl) | 27.624 |
| Tetracosanoic acid, tert-butyldimethylsilyl ester | 27.73 |
| Stigmast-5-en-3-ol, oleate | 27.891 |
| 9,19-Cyclolanost-23-ene-3,25-diol, 3-acetate, (3.beta.,23E) | 28.063 |
| Hexacosanoic acid, trimethylsilyl ester | 28.165 |
| alpha.-Tocopherol (vitamin E), trimethysilyl derivative | 28.311 |
| SILANE, [[(3.BETA.)-CHOLEST-5-EN-3-YL]OXY]TRIMETHYL- | 28.4 |
| 9,19-Cycloergost-24(28)-en-3-ol, 4,14-dimethyl-, acetate, (3.beta.,4.alpha.,5.alpha.)- | 28.55 |
| GLUCOPYRANOSIDE-6,6-D2, METHYL-TETRAKIS-O-(TRIMETHYLSILYL) | 28.721 |
| THREONIC ACID, 2,3-BIS-O-(TRIMETHYLSILYL)-, .GAMMA.-LACTONE, D- | 28.7 |
| 6,7-DIHYDROXYCOUMARIN-.BETA.-D-GLUCOPYRANOSIDE, PENTA-TMS | 28.87 |
| Campesterol tms | 29.217 |
| TETRAHYDRODAMMARADIENOL | 29.228 |
| Stigmasterol trimethylsilyl ether | 29.436 |
| XYLOPYRANOSIDE, METHYL 2,3,4-TRIS-O-(TRIMETHYLSILYL)-, .ALPHA.-D- | 29.667 |
| Betulin | 29.769 |
| beta.-Sitosterol trimethylsilyl ether | 29.874 |
| Silane, (1,1-dimethylethyl)dimethyl(octacosyloxy) | 30.045 |
| Per-O-trimethylsilyl-(3-O-.alpha.-d-mannopyranosyl-4-O-.beta.-d-glucopyranosyl-d-glucitol) | 30.404 |
| 3.beta.,4.beta.-Bis(trimethylsiloxy)cholest-5-ene | 30.405 |
| Thymol-.beta.-d-glucopyranoside, tetrakis(O-trimethylsilyl)- | 30.592 |
| alpha.-D-Glucopyranoside, 1,3,4,6-tetrakis-O-(trimethylsilyl)-.beta.-D-fructofuranosyl 2,3,4,6-tetrakis-O-(trimethylsilyl)- | 30.686 |
| alpha.-D-Glucopyranoside, 1,3,4,6-tetrakis-O-(trimethylsilyl)-.beta.-D-fructofuranosyl 2,3,4,6-tetrakis-O-(trimethylsilyl)- | 30.79 |
| TMS ETHER OF 2-MONOOLEGLYCEROL | 31.028 |
| Per-O-trimethylsilyl-(3-O-.alpha.-d-mannopyranosyl-4-O-.beta.-d-glucopyranosyl-d-glucitol) | 31.031 |
| 14,17-Nor-3,21-dioxo-.beta.-amyrin, 17,18-didehydro-3-dehydroxy- | 31.745 |
| beta.-D-Glucopyranose, 2,3,4,6-tetrakis-O-(trimethylsilyl)-, 1-(trimethylsilyl)-1H-indole-3-acetate | 31.756 |
| D-Fructose, 3-O-[2,3,4,6-tetrakis-O-(trimethylsilyl)-.alpha.-D-glucopyranosyl]-1,4,5,6-tetrakis-O-(trimethylsilyl)- | 31.882 |
| SOLANESOL | 32.356 |
| Olean-12-en-28-oic acid, 3-(acetyloxy)-, methyl ester, (3.beta.)- | 32.992 |

**TOE-1**

| Name | RT |
|---|---|
| L-Leucine-2TMS | 6.63 |
| 1,2-Ethanediol, monoacetate | 7.008 |
| 2-BUTENEDIOIC ACID (E)-, BIS(TRIMETHYLSILYL) ESTER | 7.031 |
| Nonanoic acid, trimethylsilyl ester | 7.185 |
| Serine tritms | 7.305 |
| N,O,O-Tris(trimethylsilyl)-L-threonine | 7.687 |
| l-Aspartic acid, bis(trimethylsilyl) ester | 8.144 |
| l-Norvaline, n-butoxycarbonyl-, butyl ester | 8.349 |
| Valylvaline, N,N'-dimethyl-n-propoxycarbonyl-, butyl ester | 8.35 |
| SILANOL, TRIMETHYL-, PHOSPHATE | 8.808 |
| Malic acid, O-(trimethylsilyl)-, bis(trimethylsilyl)ester | 9.057 |
| 2,4(1H,3H)-Pyrimidinedione, dihydro-1,3-bis(trimethylsilyl)- | 9.19 |
| BUTANAL, 2,3,4-TRIS[(TRIMETHYLSILYL)OXY]-, (R*,R*)- | 9.366 |
| L-ASPARTIC ACID, N-(TRIMETHYLSILYL)-, BIS(TRIMETHYLSILYL) ESTER | 9.456 |
| Butanoic acid, 4-[bis(trimethylsilyl)amino]-, trimethylsilyl ester | 9.515 |
| Undecanoic acid, trimethylsilyl ester | 9.748 |
| Phenylalanine-2TMS | 9.81 |
| PROPANOIC ACID, 2-METHYL-2,3-BIS[(TRIMETHYLSILYL)OXY]-, TRIMETHYLSILYL ESTER | 9.914 |
| L-Threonic acid, tris(trimethylsilyl) ether, trimethylsilyl ester | 10.037 |
| L-Asparagine, N2-trimethylsilyl-, trimethylsilyl ester | 10.41 |
| D,L-Alanine, N-(tert-butyldimethylsilyl)-N-methyl-, tert-butyldimethylsilyl ester | 10.561 |
| 3,8-Dioxa-2,9-disiladecane, 2,2,9,9-tetramethyl-5,6-bis[(trimethylsilyl)oxy] | 10.61 |
| Glutamine, tris(trimethylsilyl) | 10.65 |
| Amine, N,N,N-tris((trimethylsilyloxy)ethyl) | 10.717 |
| PENTITOL, 3-DESOXY-TETRAKIS-O-(TRIMETHYLSILYL) | 10.777 |
| Gluconic acid, 2-methoxime, tetra(trimethylsilyl)-, trimethylsilyl ester | 11.131 |
| L-Asparagine, N,N2-bis(trimethylsilyl)-, trimethylsilyl ester | 11.291 |
| 1,2-Epoxy-3,4-dihydroxycyclohexano[a]pyrene | 11.357 |
| d-Ribose, 2,3,4,5-tetrakis-O-(trimethylsilyl)-, O-methyloxime | 11.451 |
| ERYTHRITOL-1-D1, TETRAKIS-O-(TRIMETHYLSILYL) | 11.542 |
| Xylitol, 1,2,3,4,5-pentakis-O-(trimethylsilyl) | 11.643 |
| Ribitol, 1,2,3,4,5-pentakis-O-(trimethylsilyl)- | 11.776 |
| Xylitol, 1,2,3,4,5-pentakis-O-(trimethylsilyl) | 11.783 |
| L-Lysine-4TMS | 11.88 |
| l-(-)-Arabitol, pentakis(trimethylsilyl) ether | 11.977 |
| D-GLUCITOL, 1,2,3,4,5,6-HEXAKIS-O-(TRIMETHYLSILYL) | 12.154 |
| Ribonic acid, 2,3,4,5-tetrakis-O-(trimethylsilyl)-, trimethylsilyl ester | 12.28 |
| Ribonic acid, 2,3,4,5-tetrakis-O-(trimethylsilyl)-, trimethylsilyl ester | 12.425 |
| Ribonic acid, 2,3,4,5-tetrakis-O-(trimethylsilyl)-, trimethylsilyl ester | 12.521 |
| Azelaic acid, bis(trimethylsilyl) ester | 12.575 |
| L-Valine, N-(trimethylsilyl)-, trimethylsilyl ester | 12.858 |
| n-Tridecanoic acid, trimethylsilyl ester | 12.867 |
| 1,1'-BIPHENYL]-4-CARBOXYLIC ACID, TRIMETHYLSILYL ESTER | 12.95 |
| Tetradecanoic acid, trimethylsilyl ester | 13.065 |
| D-Fructose, 1,3,4,5,6-pentakis-O-(trimethylsilyl)-, O-methyloxime | 13.21 |
| 2,3,4,6-Tetra-O-trimethylsilyl-1-[1,7-dicarba-closo-dodecaboran-(12)-1-yl]-D-glucopyranose | 13.256 |
| 2-Thiobarbituric acid, S-trimethylsilyl-, bis(trimethylsilyl) ether | 13.424 |
| D-FRUCTOSE, O-METHYLOXIM, PENTAKIS-O-(TRIMETHYLSILYL) | 13.582 |
| D-Fructose, 1,3,4,5,6-pentakis-O-(trimethylsilyl)-, O-methyloxime | 13.688 |
| GALACTOSE OXIME 6TMS | 13.841 |
| Glucose oxime hexakis(trimethylsilyl) | 14.019 |
| Acetamide, N-[2-[5-methoxy-1-(trimethylsilyl)-1H-indol-3-yl]ethyl]-N-(trimethylsilyl) | 14.109 |
| D-GLUCITOL, 1,2,3,4,5,6-HEXAKIS-O-(TRIMETHYLSILYL)- | 14.131 |
| D-Glycero-D-gulo-Heptonic acid, 2,3,5,6,7-pentakis-O-(trimethylsilyl)-, .gamma.-lactone | 14.237 |
| D-Mannopyranose, 1,2,3,4,6-pentakis-O-(trimethylsilyl) | 14.661 |
| cis-9-Hexadecenoic acid, trimethylsilyl ester | 14.818 |
| Hexadecanoic acid, trimethylsilyl ester | 15.005 |
| 1,2,3,4,6-PENTAKIS-O-(TRIMETHYLSILYL)HEXOPYRANOSE | 15.132 |
| GLUCARIC ACID, 2,3,4,5-TETRAKIS-O-(TRIMETHYLSILYL)-, BIS(TRIMETHYLSILYL) ESTER | 15.271 |
| Tetradecanoic acid, dimethyl(isopropyl)silyl ester | 15.316 |
| Myo-Inositol, 1,2,3,4,5,6-hexakis-O-(trimethylsilyl)- | 15.672 |
| TRIMETHYL({2,3,4,5,6-PENTAKIS[(TRIMETHYLSILYL)OXY]CYCLOHEXYL}OXY)SILANE | 15.827 |
| Arabino-hexaric acid, 3-deoxy-2,4,5-tris-O-(trimethylsilyl)-, bis(trimethylsilyl) ester | 15.903 |
| Heptadecanoic acid, trimethylsilyl ester | 15.905 |
| Glucose oxime hexakis(trimethylsilyl) | 16.165 |
| OELSAEURE, TRIMETHYLSILYLESTER | 16.564 |
| OLEIC ACID, TRIMETHYLSILYL ESTER | 16.625 |
| Octadecanoic acid, trimethylsilyl ester | 16.782 |
| 9,12-Octadecadienoic acid (Z,Z)-, trimethylsilyl ester | 16.945 |
| Hexadecanoic acid, tert-butyldimethylsilyl ester | 17.108 |
| 9,12-Octadecadienoic acid (Z,Z)-, trimethylsilyl ester | 17.325 |
| Nonadecanoic acid, trimethylsilyl ester | 17.619 |
| 2-O-Glycerol-.alpha.-d-galactopyranoside, hexa-TMS | 17.684 |
| Ferulic acid, trimethylsiloxy, trimethylsilyl ester | 17.783 |
| Isopimaric acid TMS | 17.908 |
| Squalene | 17.995 |
| Ricinoleic acid, trimethylsiloxy, trimethylsilyl ester | 18.223 |
| cis-13-Eicosenoic acid, trimethylsilyl ester | 18.3 |

| Name | RT |
|---|---|
| Eicosanoic acid, trimethylsilyl ester | 18.443 |
| Myo-Inositol, 1,3,4,5,6-pentakis-O-(trimethylsilyl)-, bis(trimethylsilyl) phosphate | 18.575 |
| 17-Octadecynoic acid, tert-butyldimethylsilyl ester | 18.582 |
| trans-9-Octadecenoic acid, tert-butyldimethylsilyl ester | 18.672 |
| Uridine, 2',3',5'-tris-O-(trimethylsilyl) | 18.804 |
| Octadecanoic acid, tert-butyldimethylsilyl ester | 18.825 |
| ANDROSTANE, SILANE DERIV. | 19.035 |
| Heneicosanoic acid, trimethylsilyl ester | 19.389 |
| alpha.-D-Glucopyranoside, 1,3,4,6-tetrakis-O-(trimethylsilyl)-.beta.-D-fructofuranosyl 2,3,4,6-tetrakis-O-(trimethylsilyl) | 19.697 |
| Nonadecanoic acid, tert-butyldimethylsilyl ester | 19.874 |
| Hexadecanoic acid, 2,3-bis[(trimethylsilyl)oxy]propyl ester | 20.035 |
| Per-O-trimethylsilyl-(3-O-.alpha.-d-mannopyranosyl-4-O-.beta.-d-glucopyranosyl-d-glucitol) | 20.268 |
| Docosanoic acid, trimethylsilyl ester | 20.547 |
| Ricinoleic acid, trimethylsiloxy, trimethylsilyl ester | 20.717 |
| D-Turanose, heptakis(trimethylsilyl) | 21.007 |
| alpha.-D-Glucopyranoside, 1,3,4,6-tetrakis-O-(trimethylsilyl)-.beta.-D-fructofuranosyl 2,3,4,6-tetrakis-O-(trimethylsilyl) | 21.389 |
| Tetracosanoic acid, trimethylsilyl ester | 21.968 |
| D-Turanose, heptakis(trimethylsilyl) | 22.162 |
| D-Turanose, heptakis(trimethylsilyl) | 22.352 |
| L-Methionine-2TMS | 22.611 |
| D-Fructose, 3-O-[2,3,4,6-tetrakis-O-(trimethylsilyl)-.alpha.-D-glucopyranosyl]-1,4,5,6-tetrakis-O-(trimethylsilyl) | 22.989 |
| D-Turanose, heptakis(trimethylsilyl) | 23.303 |
| D-Turanose, heptakis(trimethylsilyl) | 23.511 |
| Tetracosanoic acid, trimethylsilyl ester | 23.765 |
| Cholest-5-en-3-ol (3.beta.)-, carbonochloridate | 25.289 |
| Ricinoleic acid, trimethylsiloxy, trimethylsilyl ester | 26.111 |
| Tricosanoic acid, tert-butyldimethylsilyl ester | 26.602 |
| Tocopherol-.beta.-tms-derivative | 26.885 |
| Melibiose, octakis(trimethylsilyl) | 27.349 |
| STIGMAST-5-EN-3-OL, (3.BETA.) | 27.546 |
| D-Glucose, 4-O-[2,3,4,6-tetrakis-O-(trimethylsilyl)-.beta.-D-galactopyranosyl]-2,3,5,6-tetrakis-O-(trimethylsilyl) | 27.624 |
| Tetracosanoic acid, tert-butyldimethylsilyl ester | 27.731 |
| Stigmast-5-en-3-ol, oleate | 27.891 |
| 9,19-Cyclolanost-23-ene-3,25-diol, 3-acetate, (3.beta.,23E) | 28.064 |
| Hexacosanoic acid, trimethylsilyl ester | 28.165 |
| SILANE, [[(3.BETA.)-CHOLEST-5-EN-3-YL]OXY]TRIMETHYL- | 28.401 |
| 9,19-Cycloergost-24(28)-en-3-ol, 4,14-dimethyl-, acetate, (3.beta.,4.alpha.,5.alpha.) | 28.55 |
| D-Glucose, 4-O-[2,3,4,6-tetrakis-O-(trimethylsilyl)-.beta.-D-galactopyranosyl]-2,3,5,6-tetrakis-O-(trimethylsilyl)- | 28.697 |
| THREONIC ACID, 2,3-BIS-O-(TRIMETHYLSILYL)-, .GAMMA.-LACTONE, D- | 28.7 |
| 6,7-DIHYDROXYCOUMARIN-.BETA.-D-GLUCOPYRANOSIDE, PENTA-TMS | 28.868 |
| Campesterol tms | 29.219 |
| TETRAHYDRODAMMARADIENOL | 29.23 |
| Stigmasterol trimethylsilyl ether | 29.439 |
| XYLOPYRANOSIDE, METHYL 2,3,4-TRIS-O-(TRIMETHYLSILYL)-, .ALPHA.-D- | 29.667 |
| Betulin | 29.767 |
| beta.-Sitosterol trimethylsilyl ether | 29.875 |
| Silane, (1,1-dimethylethyl)dimethyl(octacosyloxy) | 30.044 |
| Per-O-trimethylsilyl-(3-O-.alpha.-d-mannopyranosyl-4-O-.beta.-d-glucopyranosyl-d-glucitol) | 30.401 |
| alpha.-D-Glucopyranoside, 1,3,4,6-tetrakis-O-(trimethylsilyl)-.beta.-D-fructofuranosyl 2,3,4,6-tetrakis-O-(trimethylsilyl) | 30.592 |
| alpha.-D-Glucopyranoside, 1,3,4,6-tetrakis-O-(trimethylsilyl)-.beta.-D-fructofuranosyl 2,3,4,6-tetrakis-O-(trimethylsilyl) | 30.685 |
| alpha.-D-Glucopyranoside, 1,3,4,6-tetrakis-O-(trimethylsilyl)-.beta.-D-fructofuranosyl 2,3,4,6-tetrakis-O-(trimethylsilyl) | 30.789 |
| TMS ETHER OF 2-MONOOLEGLYCEROL | 31.02 |
| Per-O-trimethylsilyl-(3-O-.alpha.-d-mannopyranosyl-4-O-.beta.-d-glucopyranosyl-d-glucitol) | 31.03 |
| 14,17-Nor-3,21-dioxo-.beta.-amyrin, 17,18-didehydro-3-dehydroxy | 31.733 |
| D-Fructose, 3-O-[2,3,4,6-tetrakis-O-(trimethylsilyl)-.alpha.-D-glucopyranosyl]-1,4,5,6-tetrakis-O-(trimethylsilyl) | 31.883 |
| SOLANESOL | 32.355 |
| Olean-12-en-28-oic acid, 3-(acetyloxy)-, methyl ester, (3.beta.) | 32.99 |

**Supplementary Table S4: List of primers used for PCR and qRT-PCR**

| | |
|---|---|
| *AmA1*F | 5’-CACCATGGCGGGATTACCAGTG-3’ |
| *AmA1*R | 5’-CAAGGAAGAACCCTCTTGTTTCC-3’ |
| *AmA1RT*F | 5’- GAGATAATAGAATTGGGATCCAACAAC-3’ |
| *AmA1RT*R | 5’- CCAAAGAGACGACTTACAACGTTTT-3’ |
| *CHSF* | 5’-TGGGCCTGGGCTTACAATC-3’ |
| *CHSR* | 5’- CTTTGGGCCGGGCTTAA-3’ |
| *CHIF* | 5’-GCGGAGGAGTTGACGGATT-3’ |
| *CHIR* | 5’-TTCTCAAAGGGACCCGTAACG-3’ |
| *F3HF* | 5’-TATTCAAGGTGGCCGGACAA-3’ |
| *F3HR* | 5’-CAGCAGTTTGCATGCCAAGT-3’ |
| *DFRF* | 5’-TTATCGGCTCCTGGTTGGT-3’ |
| *DFRR* | 5’-TGTCCGCTTTCGGTAGTTC-3’ |
| *ANSF* | 5’-GCGTCCCGAACTCCATCAT-3’ |
| *ANSR* | 5’-CTTGCCGTTGCTGAGGATCT-3’ |
| *UFGTF* | 5’-GCCGCCACTCCAAACG-3’ |
| *UFGTR* | 5’- CATTCCTGGGATTACTTTCAGCTT-3’ |
| *Actin FL*F | 5’-GGGATTGATTCTCCTTTCTAACACT-3’ |
| *Actin FL*R | 5’- TGATCTCATTCATGCCGGTATC -3’ |
| *Actin RT*F | 5’-CTCCCCTAATGAGTGTGATGTGAT-3’ |
| *Actin RT*R | 5’- GAGCCCCATGAGAACATTACCA-3’ |

*AmA1*: Amaranth seed albumin 1; *CHS:* chalcone synthase; *CHI:* chalcone flavanone isomerase; *F3H:* flavanone 3-hydroxylase; *DFR:* dihydroflavonol 4-reductase; *ANS:* anthocyanidin synthase; *UFGT:* UDP-glucose flavonoid 3-O-glucosyl transferase; F: forward primer; R: reverse primer; RT: real time; FL: full length
